# Comparative transcriptomics reveal tissue level specialization towards diet in prickleback fishes

**DOI:** 10.1007/s00360-021-01426-1

**Published:** 2022-01-25

**Authors:** Michelle J. Herrera, Joseph Heras, Donovan P. German

**Affiliations:** 1 Department of Ecology and Evolutionary Biology, University of California, Irvine, 321 Steinhaus Hall, Irvine, CA 92697-2525 USA; 2 Department of Biology, California State University, San Bernardino, 5500 University Parkway, San Bernardino, CA 92407 USA

**Keywords:** Digestion, Transcriptomics, Gut, Intestine, Prickleback fish, Physiology

## Abstract

Beyond a few obvious examples (e.g., gut length, amylase activity), digestive and metabolic specializations towards diet remain elusive in fishes. Thus, we compared gut length, δ^13^C and δ^15^N signatures of the liver, and expressed genes in the intestine and liver of wild-caught individuals of four closely-related, sympatric prickleback species (family Stichaeidae) with different diets: *Xiphister mucosus* (herbivore), its sister taxon *X. atropurpureus* (omnivore), *Phytichthys chirus* (omnivore) and the carnivorous *Anoplarchus purpurescens*. We also measured the same parameters after feeding them carnivore or omnivore diets in the laboratory for 4 weeks. Growth and isotopic signatures showed assimilation of the laboratory diets, and gut length was significantly longer in *X. mucosus* in comparison to the other fishes, whether in the wild, or in the lab consuming the different diets. Dozens of genes relating to digestion and metabolism were observed to be under selection in the various species, but *P. chirus* stood out with some genes in the liver showing strong positive selection, and these genes correlating with differing isotopic incorporation of the laboratory carnivore diet in this species. Although the intestine showed variation in the expression of hundreds of genes in response to the laboratory diets, the liver exhibited species-specific gene expression patterns that changed very little (generally <40 genes changing expression, with *P. chirus* providing an exception). Overall, our results suggest that the intestine is plastic in function, but the liver may be where specialization manifests since this tissue shows species-specific gene expression patterns that match with natural diet.

**Supplementary Information:**

The online version contains supplementary material available at 10.1007/s00360-021-01426-1.

## Introduction

Vertebrates consume a large array of food items, and their digestive tracts reflect a complexity influenced by diet and genetics (Karasov and Douglas 2013; Karasov and Martínez del Rio 2007). Because different vertebrate taxa consume different diets, there tends to be variation in the morphology, size, pH, and enzyme biochemistry of their digestive systems (German 2011; Karasov and Martínez del Rio 2007; Starck 2005; Stevens and Hume 1995). As the supply organ of nutrients to an animal, the digestive system can also be plastic in its responses to dietary perturbations, ranging from changes in gene expression (De Santis et al. 2015a, b; Gawlicka and Horn 2006; He et al. 2013; Kim et al. 2014; Król et al. 2016; Le et al. 2019; Parris et al. 2019; Wang et al. 2015), to changes in digestive tract size (Fuentes and Cancino 1990; German and Horn 2006; He et al. 2013; Leigh et al. 2018), digestive enzyme activities (German et al. 2004, 2010; Harpaz and Uni 1999; He et al. 2013), and nutrient transporter activity (Buddington et al. 1987; Day et al. 2014; Verri et al. 2017).

Although plasticity of digestive tract function is well investigated on many levels in model terrestrial systems (Karasov and Douglas 2013; Karasov and Martínez del Rio 2007), and in a handful of (mostly carnivorous) aquaculture species (reviewed in Grossel et al. 2011), plasticity of fish digestive systems remains poorly investigated, particularly in an evolutionary context of dietary specialization (German and Horn 2006; German et al. 2004, 2010). Fishes compose the largest vertebrate group, and yet, it is not clear what dietary specialization means on the gut level for various taxa (German 2011; German et al. 2016), beyond what is not tolerated in aquaculture feed formulation (e.g., Król et al. 2016). For instance, in terms of ecomorphology, the oral jaws of cichlid fishes show incredible diversity leading to resource specialization in various species, yet the pharyngeal jaws of these same species show marked generality and plasticity, suggesting that the true masticatory apparatus of the oral cavity (i.e., the pharyngeal jaws) maintains the ability to process a wide-array of ingested foods (Gunter et al. 2013; Liem 1973; Meyer 2015; Stiassny and Jensen 1987; Burress et al. 2020). The plasticity displayed in some fish digestive systems suggests that the guts of some fish species may be equally as generalized and able to respond to dietary shifts (e.g., German et al. 2010; Harpaz and Uni 1999; Leigh et al. 2018; Wang et al. 2015) but there are exceptions (e.g., German and Horn 2006; German et al. 2004). Thus, we do not fully understand the general principles of fish nutritional physiology and what constitutes dietary specialization for them. To address this research gap, we took a systems approach by integrating nutritional physiology and transcriptomics to better understand digestive system plasticity in response to dietary perturbations. In addition to changes in gene expression, fishes can certainly have mutational or gene copy number differences that can help explain physiological and biochemical variation among them, thus highlighting the importance of a modern molecular approach, like transcriptomics (German et al. 2016; Heras et al. 2020; Betancor et al. 2018). RNA-seq using the Illumina high-throughput sequencing platform can provide whole de novo transcriptome information, gene functional information, and the molecular mechanisms of biological processes, including those related to digestion and metabolism, without requiring a reference genome (Martin et al. 2016; Martin and Król 2017; Qi et al. 2011).

**Fig. 1:**
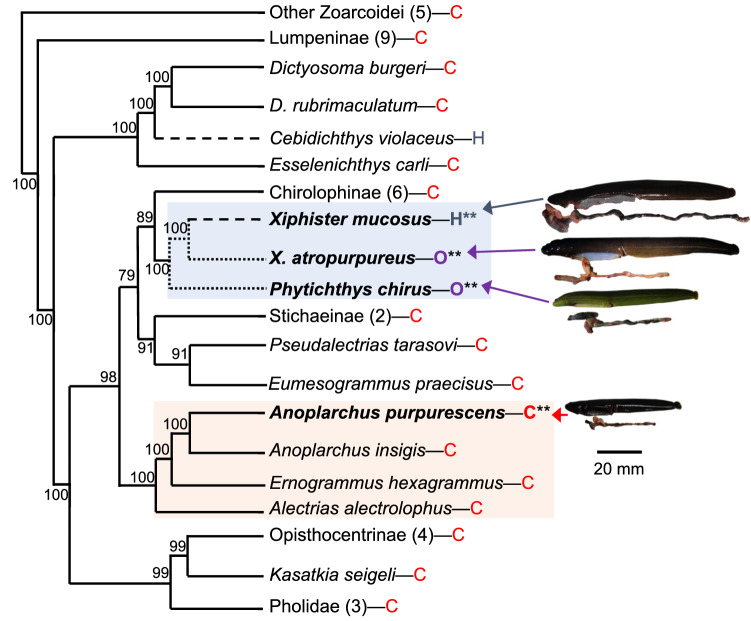
Phylogenetic relationships of the polyphyletic family Stichaeidae based on 2100 bp of *cytb*, *16 s*, and *tomo4c4* genes** (**Kim et al. 2014**).** Bayesian posterior probabilities are indicated on nodes. Studied taxa are bolded, and photos are shown with their digestive systems beneath their bodies. Note the differences in gut size. *H* herbivory, *O* omnivory, *C* carnivory. Evolution of herbivory (— — — —) and omnivory (…………) are shown. Numbers in parentheses show number of taxa evaluated at that branch. Boxes highlight some of the alleged families or subfamilies within the polyphyletic family Stichaeidae, with Xiphisterinae (top), and Alectriinae (bottom) highlighted

For this study, we used prickleback fishes (Family Stichaeidae) since they provide an excellent system in which to investigate fish nutritional physiology. With dietary variation, ontogenetic dietary shifts, convergent evolution of herbivory, and sister taxa with different diets, the Stichaeidae offers multiple opportunities to understand how fishes thrive on their specific diets and the mechanisms underlying digestive specialization (Fig. 1; German et al. 2015; Kim et al. 2014). Moreover, there is a rich literature developing on the digestive physiology (German et al. 2004, 2014, 2015; German and Horn 2006; Kim et al. 2014) and genomics (German et al. 2016; Heras et al. 2020) of these species, providing ample opportunity to test for dietary specialization. We studied four closely-related, intertidal stichaeid species with different diets: *Xiphister mucosus* (herbivore), *X. atropurpureus* (omnivore), *Phytichthys chirus* (omnivore), and *Anoplarchus purpurescens* (carnivore). Thorough gut content analyses of all target species dating back approximately four decades confirm that *X. mucosus* has greater than 98% algal material composing their diets, and the omnivores have at least 50% algae composing theirs (Horn et al. 1982; Horn et al. 1986a, b; Setran and Behrens 1993; Chan et al. 2004; German and Horn 2006; German et al. 2014; German et al. 2015). The herbivorous and omnivorous species clearly have greater carbohydrate digestive capacity and positive allometry of gut length in comparison to the carnivores (German et al. 2004, 2014, 2015, 2016). All of these species are sympatric, meaning they experience similar environmental conditions in their intertidal habitat, with diet being one of the only differences among them in the wild (German and Horn 2006; German et al. 2004, 2015).

This study had two main objectives: (1) an evaluation of differences in diet, gut length, and genes under selection from the digestive system and liver among wild-caught fishes with different diets (Table 1); and (2) an evaluation of gut length, gene expression patterns of the digestive system and liver, growth rates, and metabolic rates of the same species experiencing dietary shifts in the laboratory (Table 2). Objective one allows us to examine baseline differences among species with different diets in the wild, whereas objective two allows us to see how flexible these parameters are in the face of laboratory dietary perturbations. We focused on gut length (i.e., the length of the entire digestive system; German and Horn 2006), which can show plasticity, and tends to be longer in fishes consuming lower quality foods, such as algae (Fig. 1; Farrell et al. 2011; German and Horn 2006; Davis et al. 2013). Although detailed gut content analyses have been performed on pricklebacks in previous investigations (German and Horn 2006; German et al. 2004, 2015), there are limited studies using stable isotopic analyses to examine trophic relationships in these species (Saba 2004), and thus, we measured the δ^13^C and δ^15^N signatures of the fishes’ livers from the wild to discern dietary differences among wild-caught fishes (Guelinckx et al. 2007). We also used liver stable isotopic signatures to confirm that the laboratory-reared fishes were assimilating the assigned diets. In the laboratory, as measures of performance on the different diets, we measured growth rate across a four-week feeding trial, and the routine metabolic rates of the fish to observe whether different diets altered their metabolic rates (Reardon and Chapman 2010).

**Table 1:** Predictions for wild-caught fishes

Wild fishes	*Xiphister mucosus* (H)	*X. atropurpureus* (O)	*Phytichthys chirus* (O)	*Anoplarchus purpurescens* (C)
Relative Gut Length	Longest	Moderate	Moderate	Shortest
δ^13^C signature	Less enriched	Moderately enriched	Moderately enriched	Highly enriched
δ^15^N signature	Less enriched	Less enriched	Moderately enriched	High
Genes under positive selection	High in genes for carbohydrate degradation and carboxyl ester lipase	High in genes for carbohydrates and protein digestion and metabolism	High in genes for carbohydrate and protein digestion and metabolism	High in genes for protein metabolism

*H* herbivore, *O* omnivore, *C* carnivore

**Table 2:** Predictions for fishes fed different diets in the laboratory relative to wild-caught fishes

Lab fishes	Laboratory omnivore diet	Laboratory carnivore diet
Relative gut length	Moderate	Smallest
Growth	Moderate	Largest
Metabolic rate	Moderate	Highest
δ^13^C signature	More enriched	More enriched
δ^15^N signature	More enriched	More enriched
Relative gene expression	Elevated expression of genes involved in carbohydrate and protein digestion and metabolism	Elevated expression of genes involved in protein digestion and metabolism, gluconeogenesis, and lipid synthesis

Based on previous investigations of prickleback digestive physiology, genetics of amylase genes, and a genome of the herbivorous *Cebidichthys violaceus*, we expected to find signatures of dietary specialization in the different fish tissues (Tables 1, 2; German et al. 2014, 2004, 2015; German and Horn 2006; Heras et al. 2020). Beyond the intestinal tissues, in which we predicted to observe evidence of selection on digestive enzyme genes (Table 1), and differences in gene expression in response to laboratory diet shifts (Table 2), the liver may provide insight into metabolic pathways favored by the different species with different diets, and whether those can shift when the animals are consuming different nutrient loads in the laboratory (Yang et al. 2017; Merkin et al. 2012). Thus, we investigated how specialized these animals are for their respective diets and provide insight into the underpinnings of their abilities (or lack thereof) to use a broader base of resources than they would naturally.

## Materials and methods

### Fish capture and tissue preparation of wild individuals

Juveniles of *X. mucosus*^*H*^*, X. atropurpureus*^*O*^*, P. chirus*^*O*^*,* and *A. purpurescens*^*C*^ (112 individuals total) were collected by hand and dipnet in June 2016 at low tide from rocky intertidal habitats on San Juan Island (Dead Man Bay 48.51°N, 123.14°W and Cattlepoint; 48.45°N, 122.96°W). Superscript letters denote their natural diets: H = herbivore, O = omnivore, and C = carnivore. Fifteen juveniles of each species were transported live in seawater to Friday Harbor Laboratories (Friday Harbor, WA) where they were placed in wet table aquaria with flow through seawater (held at approximately 13 °C) to be used in a feeding experiment. The remaining individuals of each species (at least 11 of each species), abbreviated as WF (wild-caught fish), were euthanized with an overdose of tricaine methanesulfonate (MS-222 in 1 gL^−1^ seawater), measured [standard length (mm)], weighed (g), and dissected on a cutting board kept on ice (4 °C) within 4 h of collection. The digestive system of each fish was removed by cutting at the esophagus and at the anus. The gut was removed, uncoiled, and the total gut length (mm) measured as the distance from the pyloric sphincter to the distal-most end of the intestine. The measured digestive systems were used to calculate relative gut length, which is the ratio of gut length/standard length (German and Horn 2006). The liver, stomach, and pyloric ceca were excised. The intestine was divided into three sections of equal length and the sections were designated as the proximal, mid, or distal intestine. The contents of the stomach and intestine were emptied into their own vials. Approximately 100 mg of each of the tissues were immediately placed in 0.5 mL centrifuge vials containing RNAlater, and stored overnight at 4 °C, and subsequently transferred to a −80 °C freezer for storage until further processing (less than 1 week). The remaining portions of the tissues, stomach, and intestinal contents were frozen on dry ice and transferred to −80 °C freezer for storage for stable isotopic analysis, digestive enzyme activity assays, and other uses.

### Food preparation and feeding experiment

The remaining 15 individuals of *X. mucosus*^*H*^*, X. atropurpureus*^*O*^*, P. chirus*^*O*^*,* and *A. purpurescens*^*C*^ were individually placed in cubicles (approximately 1.5-L in volume) within wet table flow-through aquaria and used for a feeding experiment. Each individual fish was anesthetized (0.1 gL^−1^ MS-222), measured and weighed, and assigned to a carnivore, abbreviated as LC (Lab Carnivore), or omnivore diet, abbreviated as LO (Lab Omnivore), at the start of the experiment. All individuals of *X. atropurpureus*^*O*^ and *P. chirus*^*O*^ were fed the LC diet, as none would consume the LO diet in the laboratory. The fishes were acclimated to laboratory conditions and the formulated diet for two weeks. Fresh thalli of the algal species *Ulva lobata* (Chlorophyta), *Mazzaella splendens* (Rhodophyta), and *Porphyra* sp. (Rhodophyta), all of which are common in the diets of *X. mucosus*^*H*^*, X. atropurpureus*^*O*^*, P. chirus*^*O*^ (Horn et al. 1986a, b; German et al. 2004, 2014, 2015), were collected from the intertidal zone from which the fish were collected, and initially dried in the sun. Sundried algae were transferred into a 60 °C drying oven and dried overnight. Flatfish (several species) were collected by seining and otter trawl around San Juan Island, WA, and were mortalities from fish surveys. Dead flatfish were decapitated and skinned to produce fillets, which were dried to a constant weight at 60 °C. Vitamin and mineral premixes were obtained from Zeigler Bros. Aquafeed, whereas other ingredients (Fish oil, casein, soybean meal, methyl cellulose) were purchased from various vendors. Dried algae and flatfish were ground to pass through a 1-mm screen with a food processor followed by mortar and pestle. The omnivore and carnivore diets created in the laboratory were composed of varying concentrations of carbohydrates (dried algae) and protein (fish) and constant concentrations of lipids, vitamins and minerals (Table 3). Once combined, ingredients were wetted with deionized water and mixed by hand with a whisk, spread onto a cafeteria tray, and dried to a constant weight at 60 °C. The food was then crumbled and offered to the fish, which they readily consumed. Fish were fed their respective diets 2–3 times daily to satiation for 4 weeks. Feces were collected just before each feeding and the debris in each tank was siphoned out after each feeding. Proximate analyses of the diets were performed following methods of the Association of Official Analytical Chemists (AOAC International 2006). The total fat, organic matter, carbohydrate, total protein, and energetic content were quantified for the omnivore and carnivore diets (Table 3; German et al. 2010).

**Table 3:** Ingredients and chemical composition of the omnivore and carnivore diets fed to prickleback fishes in the laboratory

Diets	Omnivore diet	Carnivore diet
Ingredients (g/100 g)		
*Mazzaella splendens*	14.41	–
*Porphyra* sp.	14.42	–
*Ulva lobata*	14.42	–
Fish	43.25	86.5
Casein	2	2
Soybean meal	2	2
Oil	6	6
Methyl cellulose	1.5	1.5
Vitamin premix	1	1
Vitamin C	0.4	0.4
Mineral premix	0.6	0.6
Chemical composition		
Protein (%)	45.40	68.80
Carbohydrate (%)	19.46	2.17
Lipid (%)	12.34	11.90
Calories (Cal)	263.8	342.0
Organic matter (%)	81.40	89.14

At the conclusion of the feeding trials (4 weeks on the prescribed diets), the routine metabolic rates of each fish were measured in a respirometer and taken over a short period of time. Negative control runs (i.e., without a fish in the system) validated that there was little oxygen consumption in the system itself across the time frames of measurement (~15 min intervals). The fish were fed their normal morning feeding because we wanted to examine any instantaneous effects of the different diets on their metabolic rates. The closed chamber respirometer resembled that described by Reardon and Chapman (2010), featuring a 400 mL chamber that housed the fish, and the system contained a total of 1.9 L with a flow rate of 5 L per min set with a pump and flow meter (Supplemental Figure S1). Prickleback fishes are benthic (e.g., Ralston and Horn 1986), and the chosen flow rate did not force them to swim within the chamber. The fishes sat on the bottom of the chamber, unencumbered, for the measurements. Decreases in oxygen concentration (% O_2_ saturation) were used to estimate the rate of *V̇*O_2_ (volume of oxygen consumed per unit time) of the fish. Oxygen and temperature data were recorded every 30 s during the trial with Ocean Optics FOXY probes and thermistors, respectively. The temperature was maintained at 14 °C (± 0.2 °C) by submerging the chamber in flow-through seawater pumped directly from Friday Harbor (Supplemental Figure S1). The fish were allowed to acclimate to the chamber for at least 30 min before starting measurements. Once the O_2_ concentrations dipped below 90% saturation (approximately 15 min in the closed system), valves were manually opened, flushing the system with ambient seawater for 5 min, then manually closed again for the next measurement period. Each fish was measured three times (see Supplemental Figure S1 panel B for a representative trace). The nature of the setup did not allow us to keep the fish in the system for extended periods of time without causing significant further stress on the fish. Thus, we did not determine basal metabolic rate or specific dynamic action.

At least one full day following the metabolic rate measurements, including being fed, the fish were euthanized, measured, weighed, and dissected as described above under “Fish capture and tissue preparation of wild individuals”. Tissues were subsampled for transcriptomic and stable isotopic analyses (see below under “RNA isolation and library preparation”), with the remainder used for a separate study of digestive enzyme activity levels, gut ultrastructure, and gut microbiome. Growth of the individual fish was assessed as weight gained between the beginning and end of the experiment. There were no mortalities throughout the feeding experiment.

### Stable isotopic analyses

To assess carbon and nitrogen assimilation from the diets, we measured δ^13^C and δ^15^N signatures of liver tissue from wild-caught (four individuals for each species), LO-fed fishes (three individuals for each species) and LC-fed fishes (3 individuals for each species) and of the omnivore and carnivore diets made in the laboratory. Liver tissue and diets were dried overnight at 60 °C, and ground into powder. Approximately 0.7 mg of individual liver or diet samples were then transferred into individual 5 mm × 9 mm tin capsules (Costech Analytical Technologies). Samples were run through a Fissions NA 1500NC elemental analyzer interfaced to a ThermoFinnigan-DeltaPlus CF (Bremen, Germany) isotope ratio mass spectrometer in the Center for Isotope Tracers in Earth Science facility at UC Irvine. Stable isotope abundances are expressed in delta (δ), defined as parts per thousand (‰) relative to the standard as follows:

1 $${\delta}=[(R_{\textrm{sample}}/R_{\textrm{standard}})\,-\,1]\,(1000)$$

where *R*_sample_ and *R*_standard_ are the corresponding ratios of heavy to light isotopes (^13^C/^12^C and ^15^N/^14^N) in the sample and standard, respectively. R_standard_ for ^13^C was Vienna Pee Dee Belemnite (VPDB) limestone formation international standard. *R*_standard_ for ^15^N was atmospheric N_2_. Analyses were performed following German and Miles (2010).

### RNA isolation and library preparation

Total RNA from the tissue samples (20–50 mg) of the pyloric ceca, mid-intestine, and liver from two individual fish of each of the four species were isolated using TRIzol reagent (Thermo Fisher Scientific) following the manufacture’s protocol. We chose to evaluate more tissue types (three) as opposed to more replicates (two) of the same tissue type to get more coverage of expressed genes. We used principal component analysis (PCA) to examine the appropriateness of our replicates, which appear sufficient, as each tissue is similar to itself as opposed to being more similar to other tissues (Supplemental Figures S2–S11). All samples were extracted and prepared within days of each other. Samples were quantified (ng/μl) using an RNA Nanodrop and RNA quality was determined by Bionalyzer (RNA integrity > 7) at the UC Irvine genomics high throughput facility. Samples were prepped for Illumina sequencing using a TruSeq RNA sample prep kit (Illumina, San Diego, CA) to prepare individual cDNA libraries. Agencourt AMPure XP magnetic beads were used to re-purify the samples (Beckman Coulter Genomics, Danvers, MA). The bioanalyzer again was used to conduct a quality control check of the cDNA. The cDNA pools were normalized to 10 nM and samples were scattered, as to not have a lane or batch effect, with the pyloric ceca and mid-intestine samples run across four lanes and two runs, and a separate run containing the liver samples to constitute three paired-end 100 bp runs on a HiSeq 2500 (Illumina, San Diego, CA) by the UCI genomics high-throughput facility. All data generated were deposited into NIH Archive with accession number PRJNA738880.

### Assembly of sequence reads and gene annotation

Raw data files were filtered and trimmed with Trimmomatic v0.32 (Bolger et al. 2014) implemented in UCI’s high performance cluster (HPC), in order to make certain that trailing bases have a Phred score of a minimum of 30. Reads were then normalized to low systematic coverage to remove errors and reduce data set size using the Trinity v r2015-2.1.1 normalize_by_kmer_coverage.pl script (Haas et al. 2013). Such normalization reduces among-sample bias (Abrams et al. 2019). A *de-novo* assembly using Trinity v r2015-2.1.1 was conducted, where one “wild” individual was selected as the reference assembly and used the RNA-seq by Expectation Maximization (RSEM) package v1.2.31 to align RNA-Seq reads back to the Trinity transcripts (Grabherr et al. 2011; Li and Dewey 2011; Mandelboum et al. 2019). Annotation was conducted with Trinotate v3.0.0 annotation suite for genes under differential expression, the full transcripts of the wild individuals, and ortholog pairs and clusters. Trinotate uses TransDecoder v2.0.1 (Haas et al. 2013) to identify open reading frames (ORF), then translated and untranslated ORFs are blasted (BLASTX) against the swiss-prot database, where the best hit and gene ontologies (GO) are used for annotation. Afterwards, HMMER v3.1 tool hmmscan (Finn et al. 2011) and the Pfam-A database (Punta et al. 2012) were used to annotate protein domains for the predicted protein sequences.

### Quality check samples and biological replicates

We sequenced the transcriptomes of the liver, pyloric ceca, and mid intestine from two individuals from each diet group in each species, and we conducted a quality check to ensure our biological replicates are well correlated using the Trinity program “PtR” (Haas et al. 2013). Samples within a diet group were well correlated within their respective tissue when comparing across the liver, pyloric ceca, and mid-intestine samples. Principal component analysis (PCA) plots for each species and diet group comparing across the three different tissues we sequenced are displayed in Supplementary Figures S2–S11, showing that the tissue replicates are more similar to each other than any are to other sequenced tissues. We also conducted BatchQC v3.4 to check for any batch effects, and found that there were no strong correlations with batch as displayed in Supplementary Figure S12. Therefore, we are confident that our low sample sizes for transcriptomics are suitable for the level of analysis conducted here.

### Ortholog identification and estimation of positive selection in wild-caught fishes

Assembled sequences were masked for repetitive elements with Repeatmasker v4.0.5 (Smit 2004) with teleost fish as the query species. Using the standalone Orfpredictor v3.0 (Min et al. 2005), the open reading frame was identified and sequences with a minimum length of 60 nucleotides were used to identify orthologous pairs through Inparanoid v.4.0 (O'Brien et al. 2005) with all pairwise comparisons of the four target species (6 possible pairwise comparisons). The ortholog pairs were used to identify ortholog clusters in all four species using Quickparanoid (http://pl.postech.ac.kr/QuickParanoid/). Then, perl scripts were used to obtain ortholog clusters comparing only one sequence per four target species, with a gene seed ortholog and confidence score of 1, with no tree conflict. Orthologs clusters with one orthologous gene from each species per cluster were used for the estimation of positive selection. Protein and nucleotide sequences of the orthologs were aligned using Muscle v3.7 (Edgar 2004) and pal2nal 12.2 (Suyama et al. 2006) based on translated coding sequences. *X. mucosus*^H^ was used as a reference dataset to represent the ortholog clusters identified from all four species and, therefore, was used to annotate orthologous clusters through the Trinotate annotation suite (see “Annotation of Genes”). A perl script was used to process multiple aligned ortholog clusters into CODEML as part of the PAML v4.8a package (Yang 1997) in order to estimate positive selection. To identify genes under positive selection from all four species of wild-caught fishes, we used the following site models: M0 (one omega), M7 (beta distributed variable selective pressure), and M8 (beta distributed with positive selection) in PAML v4.8a. Models M7 (neutral) and M8 (positive selection) were compared, in which the likelihood values were used to detect positive selection using Likelihood Ratio Test (LRT). Pchisq in R v3.4.4 was used to compare LRT values of M7 and M8 with a *χ*^2^ distribution with an *α* level of significance at 0.05. We used Benjamini–Hochberg corrected *p*-values that was calculated from the *χ*^2^ distribution values and an *α* level of significance at 0.05. We viewed only the first represented gene ontology (GO) for biological processes by using REViGO (http://revigo.irb.hr/) and their corresponding omega values from the M0 PAML results.

To identify genes under positive selection, we used the omega values from the M0 PAML results. Branch selection was examined using adaptive branch-site random effects likelihood (aBSREL) test for episodic diversification (Datamonkey v 2.0 web application), and curated manually using the PAML results (Weaver et al. 2018; Smith et al. 2015; Kosakovsky Pond et al. 2011).

### Differential expression level analysis

Relative expression levels of all genes expressed in tissue types of interest were standardized to constitutively expressed Ribosomal Protein L8 using FPKM ratios calculated with eXpress (Roberts and Pachter 2013). Then, relative gene expression levels were estimated using RSEM v1.2.31 (Li and Dewey 2011), which allows for the identification of gene and isoform abundance. Therefore, the calculated gene expression can be directly used for comparing differences among individuals of the same species experiencing different diet challenges. Then, we calculated differences in the abundance of expression of each gene within individuals of the same species across the diet groups and generated heatmaps using EdgeR (Bioconductor v3.2) with an FDR < 0.001 and a dispersion value of 0.4. This was carried out for each tissue type and each species separately.

For clarity purposes, heatmaps were broken into clusters based on expression profile, which are described in Table 5 and Supplemental Materials and Methods (online).

### Statistical analyses

For study objective one, interspecific comparisons of relative gut lengths were made among the species of wild-caught fishes with analysis of covariance (ANCOVA), using body mass as a covariate. A Tukey’s honest significant difference (HSD, with an *α* = 0.05) was used to evaluate what species had longer guts than the others. δ^13^C and δ^15^N values were compared (separately) among the wild-caught fishes using ANOVA followed by a Tukey’s HSD. Ortholog comparisons and genes under positive selection are described above (see “Ortholog identification and estimation of positive selection in wild-caught fishes”). For study objective two, intraspecific comparisons of relative gut length were made among individuals fed the various diets in the laboratory and the wild-caught fish consuming their natural diets, using ANCOVA with body mass as a covariate. Outliers that were more than twice the 1.5 interquartile range were removed from statistical analyses. For *X. mucosus*^H^ and *A. purpurescens*^C^, intraspecific comparisons of growth rates and metabolic rates among individuals fed the LO and LC diets were performed with a *t*-test. Intraspecific comparisons of δ^13^C and δ^15^N values amongst wild-caught and lab-fed fishes were made with ANOVA followed by Tukey’s HSD. In addition to intra-specific comparisons of differentially expressed genes among fishes fed the different diets in the laboratory and wild-caught fishes, we also examined the similarity of expressed genes in different tissues of all of the fishes using PCA, which allowed us to qualitatively state which tissues showed the most plasticity in gene expression among the species and diet treatments. We also used the PCA vectors to estimate what made the species different from one another in terms of expressed genes. All statistics were run in R (version 3.6.0).

## Results

### Objective 1: comparisons of wild-caught fishes

#### Relative gut length

**Fig. 2:**
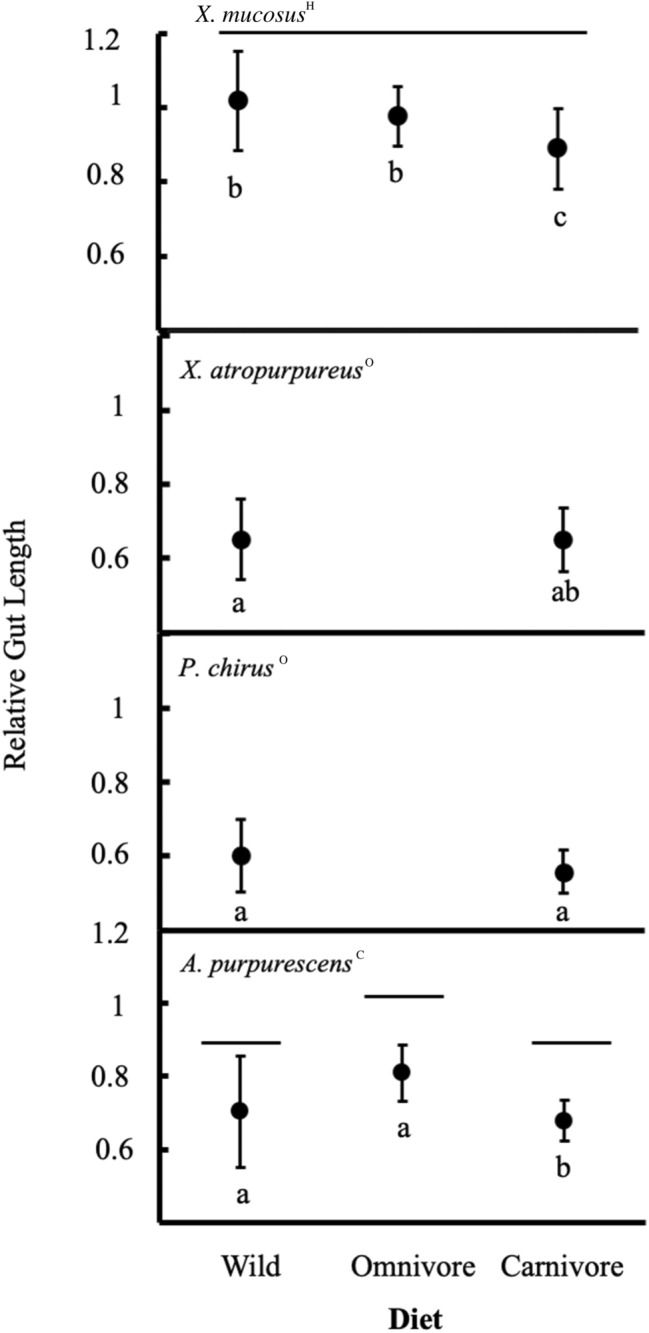
Relative gut length (gut length^.^ standard length^−1^) of wild-caught fishes, and those fed omnivore or carnivore diets in the laboratory. Top to Bottom: *X. mucosus*^H^*, X. atropurpureus*^O^*, P. chirus*^O^, and *A. purpurescens*^C^. Intraspecific comparisons of individuals on the different diets (along the *x*-axis) were made with ANCOVA (using body mass as a covariate; Supplemental Table S5), and symbols sharing a line of the same elevation are not significantly different (*P* > 0.05) from each other. No intraspecific differences were found for *X. atropurpureus*^O^ or *P. chirus*^O^, and hence, no lines are drawn. For a given dietary category (wild, omnivore, carnivore), interspecific comparisons were made (vertically) with ANCOVA (with body mass as a covariate), and symbols sharing a letter are not significantly (*P* > 0.05) different from each other

Significant differences in relative gut length were detected among wild fishes (WF) of the four species (ANCOVA species: *F*_3,44_ = 8.98, *P* < 0.001; body mass: *F*_1,44_ = 11.80, *P* < 0.01, Species x Body Mass interaction: *F*_3,44_ = 0.736, *P* = 0.537; Fig. 2), with *X. mucosus*^H^ possessing the longest guts, and no significant differences detected amongst the other species.

#### Stable isotopic analyses

**Fig. 3:**
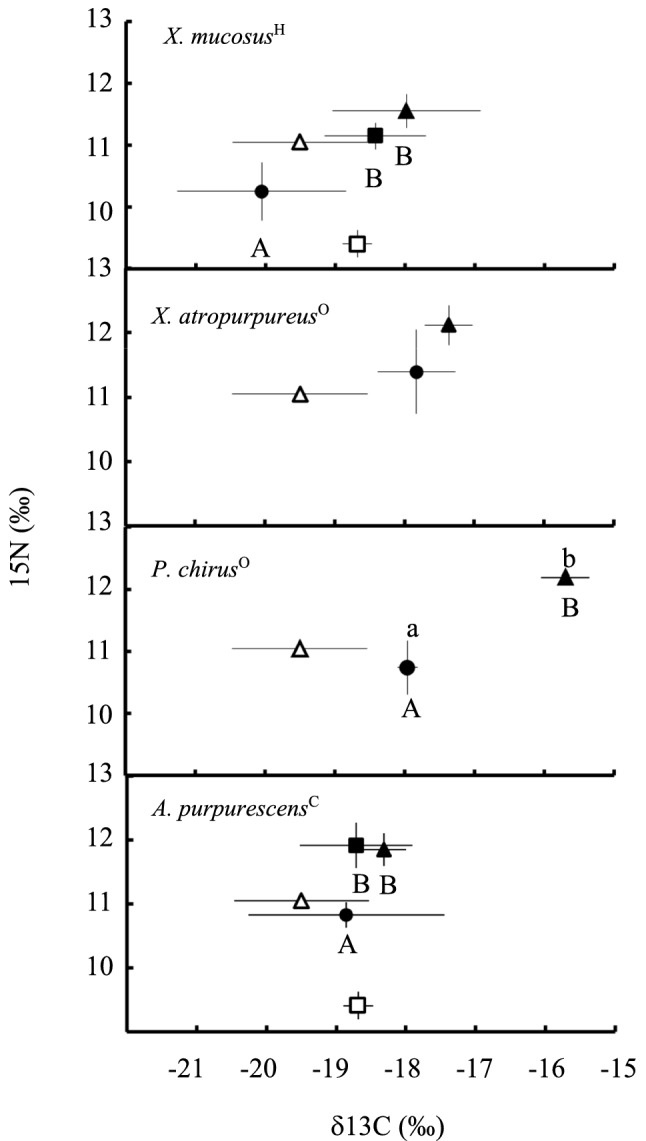
Carbon and nitrogen (‰) dual isotope plots of wild-caught fishes, and fishes fed omnivore and carnivore diets in the laboratory. Top to bottom: *X. mucosus*^*H*^, *X. atropurpureus*^O^, *P. chirus*^O^, and *A. purpurescens*^C^. Shapes indicate the following: open square: lab omnivore diet; filled square: lab omnivore fish; open triangle: lab carnivore diet; filled triangle: lab carnivore fish; filled circle: wild fishes. Values are mean ± standard deviation. Intraspecific comparisons of the fish on the different diets were made with ANOVA for each species. Significant differences (*P* < 0.05) for δ^15^N indicated with capital letters, whereas lower case letters indicate significant differences in δ^13^C values

When comparing wild-caught individuals of the different species to each other, WF *X. atropurpureus*^O^ showed enriched δ^13^C and δ^15^N signatures in comparison to WF *X. mucosus*^H^ (ANOVA: Carbon *F*_3,12_ = 4.428, *p* = 0.0258; Nitrogen *F*_3,12_ = 3.963, *p* = 0.0355, Supplemental Table S1; Figure S13), isotopically confirming that these sister taxa have different diets. Wild *P. chirus*^O^ also showed a significantly enriched δ^13^C signature compared to WF *X. mucosus*^H^ (ANOVA: Carbon *F*_3,12_ = 4.428, *p* = 0.0258, Fig. 3; Supplemental Table S1; Figure S13). Wild *A. purpurescens*^C^ had δ^13^C and δ^15^N signatures intermediate to the other species.

#### Orthologous genes and ortholog clusters

There are not as many shared orthologs among all four wild species in the liver (870 orthologs) compared to the pyloric ceca (3787 orthologs) and the mid-intestine (3267 orthologs, Table 4). The closer phylogenetically related species, such as the *X. mucosus*^H^ and *X. atropurpureus*^O^, share more orthologs with each other (e.g., pyloric ceca: 17,111 ortholog pairs) compared to a more distantly related species, such as *X. mucosus*^H^ and *A. purpurescens*^C^ (e.g., pyloric ceca: 13,079 ortholog pairs; Table 4).

**Table 4:** Orthologous gene pairs in different tissues of four closely-related prickleback fish species

Liver	*X. mucosus*^H^	*X. atropurpureus*^O^	*P. chirus*^O^	*A. purpurescens*^C^
*X. mucosus*^H^	–			
*X. atropurpureus*^O^	13,124	–		
*P. chirus*^O^	4124	4652	–	
*A. purpurescens*^C^	5311	5963	4113	–
Shared among all four species: 870				

Pyloric ceca	*X. mucosus*^H^	*X. atropurpureus*^O^	*P. chirus*^O^	*A. purpurescens*^C^
*X. mucosus*^H^	–			
*X. atropurpureus*^O^	17,111	–		
*P. chirus*^O^	13,546	16,511	–	
*A. purpurescens*^C^	13,079	15,852	15,057	–
Shared among all four species: 3787				

Mid-intestine	*X. mucosus*^H^	*X. atropurpureus*^O^	*P. chirus*^O^	*A. purpurescens*^C^
*X. mucosus*^H^	–			
*X. atropurpureus*^O^	16,166	–		
*P. chirus*^O^	13,067	17,283	–	
*A. purpurescens*^C^	12,573	16,600	16,543	–
Shared among all four species: 3267				

#### Positively selected genes

##### Liver

**Fig. 4:**
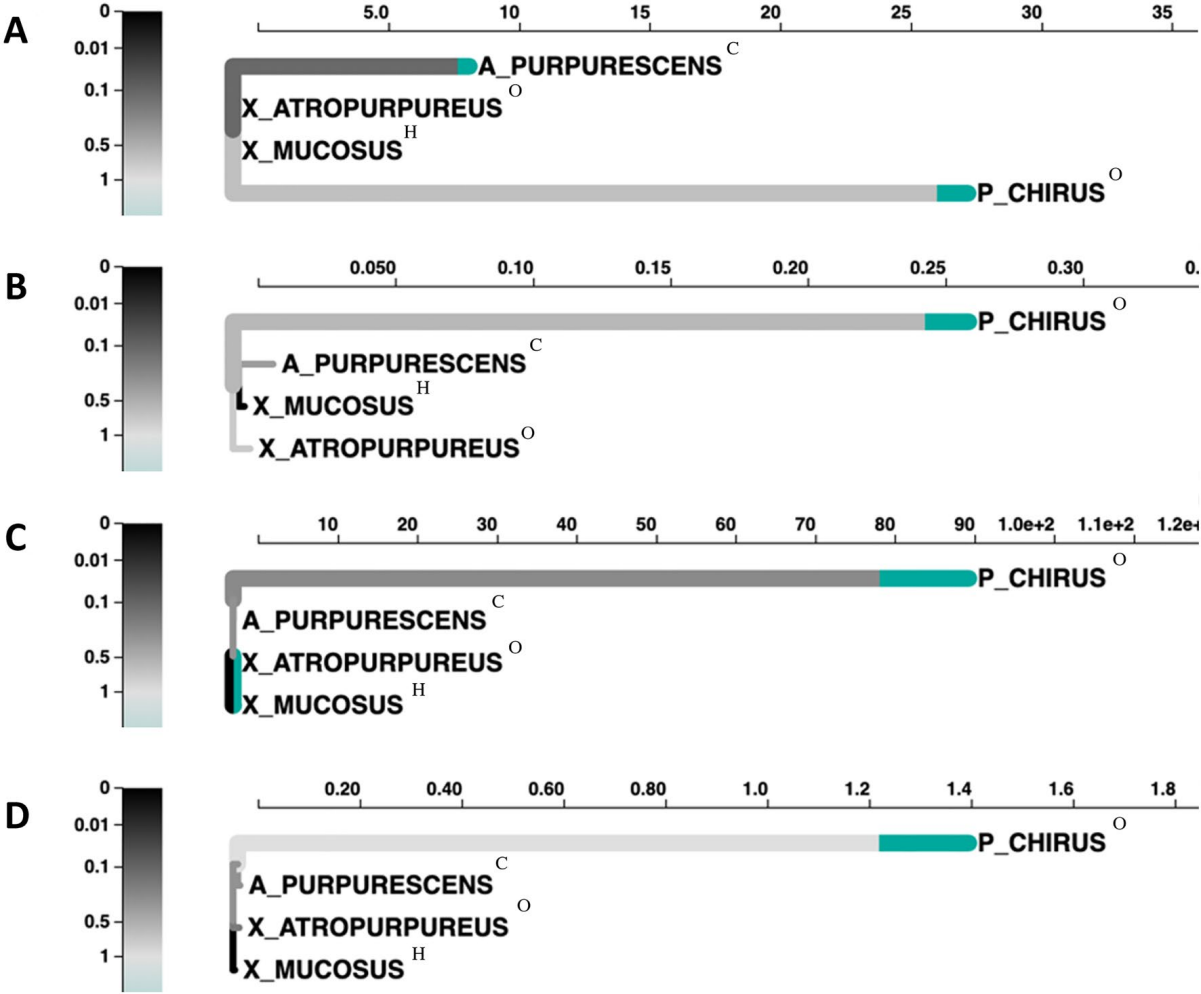
An adaptive branch-site random effects likelihood (aBSREL) test for episodic diversification phylogenetic tree constructed for various genes in the liver from four prickleback fish species: **A** Glucose-6-Phosphate 1-Dehydrogenase (G6PD), **B** fatty acid binding protein, **C** Mitochondrial import inner membrane translocase subunit (TIM21), and **D** endothelial lipase. *ω* is the ratio of nonsynonymous to synonymous substitutions. The color gradient represents the magnitude of the corresponding ω. Branches thicker than the other branches have a *p* < 0.05 (corrected for multiple comparisons) to reject the null hypothesis of all ω on that branch (neutral or negative selection only). A thick branch is considered to have experienced diversifying positive selection

We found fatty acid-binding protein, mitochondrial import inner membrane translocase subunit (TIM21), and endothelial lipase under positive selection in the liver and the transcripts contained high (100%) to medium coverage (~50%) of the full gene from the swissprot database (Supplemental Table S2, Fig. 4). Glucose-6-phosphate 1-dehydrogenase (G6PD), which is part of the pentose phosphate pathway, is under positive selection in *P. chirus*^O^. There is branch selection in G6PD for *A. purpurescens*^C^ and *P. chirus*^O^ (Fig. 4). Looking at the sites under selection, we found that many sites in the transcriptome of *P. chirus*^O^ are significantly different from the other three species. For fatty acid-binding protein, TIM21, and lipase, we find branch selection in *P. chirus*^O^ and significant selection at multiple sites in the transcriptome of *P. chirus*^O^*,* making this species stand out from the other three species (Fig. 4).

##### Pyloric ceca

The pyloric ceca featured genes involved in protein and fatty acid metabolism under positive selection, including aminopeptidase, phospholipase, elastase, and tubulin alpha chain (Supplemental Table S3, Supplemental Figure S14). The transcripts contained high to medium coverage. Serine protease 27 is under positive selection in *P. chirus*^O^ and *A. purpurescens*^C^, and there is branch selection in *A. purpurescens*^C^ as well (Supplemental Figure S14). For tubulin alpha chain, there is branch selection for *X. mucosus*^H^ (Supplemental Figure S14). There is also branch selection in *X. mucosus*^H^ and *A. purpurescens*^C^ for the protease elastase.

##### Mid-intestine

The mid-intestine featured genes involved in carbohydrate digestion and metabolism, including *α*-mannosidase, succinate dehydrogenase, and NADH dehydrogenase (Supplemental Table S4).

### Objective 2: dietary flexibility in the laboratory

#### Relative gut length

Wild *X. mucosus*^H^ had longer guts than individuals of this species fed a carnivore diet in the laboratory (LC), yet the gut lengths of the fish fed an omnivore diet in the laboratory (LO) were not statistically different from WF fish or LC fish of this species (ANCOVA diet: *F*_2,20_ = 2.32, *P* = 0.124; body mass: *F*_1,20_ = 1.53, *P* = 0.231). There were no significant differences among the relative gut lengths of WF *X. atropurpureus*^O^ (ANCOVA diet: *F*_1,21_ = 0.013, *P* = 0.912; body mass *F*_1,18_ = 3.682, *P* = 0.071) and WF *P. chirus*^O^ fish (ANCOVA diet: *F*_1,24_ = 0.623, *P* = 0.439; body mass *F*_1,21_ = 3.322, *P* = 0.08) and LC fish within each respective species. LO *A. purpurescens*^C^ had a significantly longer gut length than WF fish and LC *A. purpurescens*^C^, with the two latter groups not being significantly different in this species (ANCOVA diet: *F*_2,22_ = 3.67, *P* < 0.05; body mass: *F*_1,22_ = 5.03, *P* < 0.05, Diet × Body Mass interaction: *F*_*2,22*_ = 0.148, *P* = 0.863; Fig. 2).

Interspecific comparisons across species within a diet group showed that LO *X. mucosus*^H^ had significantly longer guts than LO *A. purpurescens*^C^ *(*ANCOVA species: *F*_1,11_ = 12.89, *P* < 0.01; body mass: *F*_1,11_ = 0.72, *P* = 0.41. Additionally, LC *X. mucosus*^H^ possessed the longest guts compared to the other three species (ANCOVA species: *F*_*3,29*_ = 5.81, *P* < 0.001; body mass: *F*_*1,29*_ = 2.56, *P* = 0.12; Fig. 2).

#### Growth rate and metabolic rate

After 4 weeks of the feeding trial, LC *X. mucosus*^H^ (16.8 ± 2.6%) exhibited a significantly higher growth rate than LO fish (5.4 ± 5.2%; *t* = 4.552, *df* = 11, *p* = 0.001; Supplemental Tables S5 and S6). There was no significant difference in growth rate between LC (22.7 ± 12.0%) and LO (20.5 ± 12.9%) *A. purpurescens*^C^ (*t* = 0.309, *df* = 11, *p* = 0.763). The growth rate of LC *P. chirus*^*O*^ individuals was 21.68 ± 8.37% and for LC *X. atropurpureus*^O^ individuals it was 11.45 ± 5.8% (Supplemental Table S6). The sizes of the fishes used in this study are in Supplemental Table S5, and it is worth noting that the individuals of *X. mucosus*^H^ were approximately double the masses of the other species.

The routine metabolic rate of LC and LO fishes of *X. mucosus*^H^ (*t* = 0.741, *df* = 9, *p* = 0.478) and *A. purpurescens*^C^ (*t* = 0.936, *df* = 9, *p* = 0.373, Supplemental Table S6) did not differ significantly within each species. There was no statistical difference among the metabolic rates of all species fed diets in the laboratory.

#### Stable isotopic analyses

The LC and LO fishes clearly assimilated the laboratory diets and are different from WF fish within the same species (Fig. 3). From the δ^15^N perspective, WF *X. mucosus*^H^ (ANOVA: *F*_2,7_ = 12.21, *p* < 0.05) and WF *A. purpurescens*^C^ (ANOVA: *p* < 0.05, *F*_2,7_ = 18.73) differed significantly from LC and LO fish within the respective species, with LC and LO groups not being statistically different (Fig. 3, and see Supplemental Table S1 for more statistical detail). LC *P. chirus*^O^ showed a statistically significant enrichment in δ^13^C (ANOVA: *F*_1,5_ = 144.2, *p* < 0.001) and δ^15^N (ANOVA: *F*_1,5_ = 29.58, *p* < 0.05) signatures of their livers relative to WF *P. chirus*^O^ fish. LC *X. atropurpureus*^O^ showed a slight enrichment in δ^15^N signatures (ANOVA: *F*_1,5_ = 3.054, *p* = 0.141) compared to the WF *X. atropurpureus*^O^ fish, but not significantly so (Fig. 3, Supplemental Table S1). When consuming the same diet in the laboratory, LO *X. mucosus*^H^ and LO *A. purpurescens*^C^ differed significantly for δ^15^N (ANOVA: *F*_1,4_ = 10.25, *p* = 0.0328, Supplemental Table S1), but not for δ^13^C (ANOVA: *F*_1,4_ = 0.216, *p* = 0.666, Supplemental Table S1). When consuming the carnivore diet in the laboratory, LC *P. chirus*^O^ stood out from the rest of the species and had statistically significant enriched δ^13^C signatures (ANOVA: *F*_3,8_ = 11.10, *p* < 0.05, Supplemental Table S1). In terms of δ^15^N, LC *P. chirus*^O^ were enriched in comparison to LC *X. mucosus*^H^ (ANOVA: *F*_3,8_ = 3.976, *p* = 0.0526, Supplemental Table S1), but no other differences were detected.

#### Relative gene expression

**Fig. 5:**
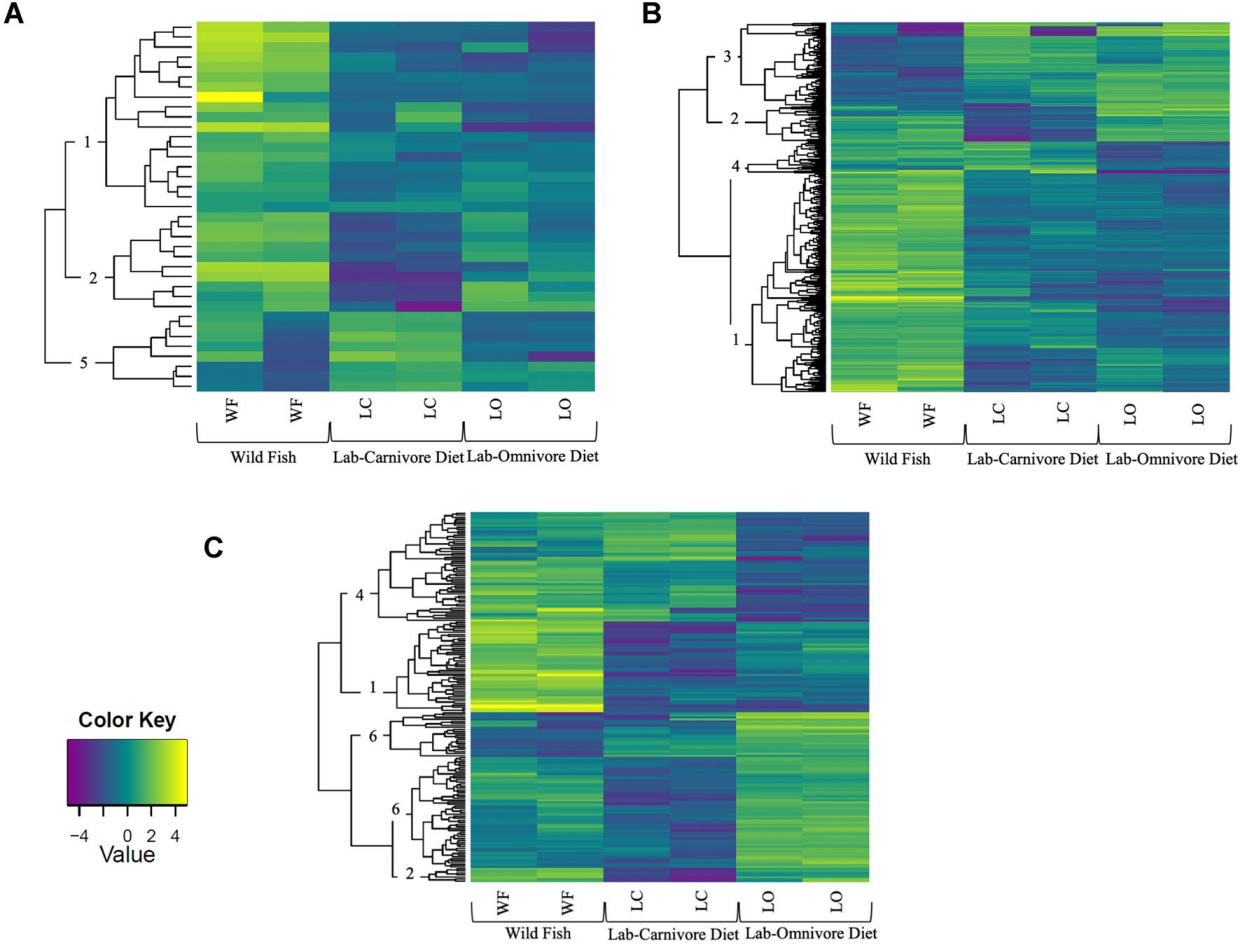
Differential gene expression depicted as heatmaps in different tissues of *X. mucosus*^H^: **A** Liver, **B** mid intestine, and **C** pyloric ceca. Yellow indicates elevated relative expression, whereas blue indicates low expression. Each row is a single gene, and genes are clustered in a dendrogram (on left of each heatmap) by similarity of expression patterns. The various clusters of genes are described in Table 4. Each column represents the gene expression in a single tissue from an individual fish, with *WF* wild-caught fish, *LO* fish fed an omnivore diet in the laboratory, and *LC* fish fed a carnivore diet in the laboratory

We used RNA-seq data of the liver, pyloric ceca and mid-intestine for each of the four species to observe the suites of genes that changed with different diets and how species respond to dietary variation. Relative expression levels of all genes expressed in tissue types of interest, which included genes involved in digestion (e.g., digestive enzymes, nutrient transporters, metabolic pathways), were analyzed. Note that we are only reporting on pathways relevant to digestion and metabolism of specific nutrient classes (Fig. 5, Table 5). If a cluster is not mentioned, yet depicted in the heatmap, then the genes within that cluster were not directly relevant to digestion and nutrient metabolism. For simplicity and space, we share heatmaps for *X. mucosus*^H^ only, and all other heatmaps, as well as details of these results, are available in the supplemental materials (Supplemental Tables S7–S9, Supplemental Figures S14–S22).

**Table 5:** Differentially expressed genes cluster definitions as presented in the heat maps in Fig. 4, and in Supplemental Figures S15–S23

Species	Cluster	Wild	Lab-carnivore diet	Lab-omnivore diet
*Xiphister mucosus*^H^ and *Anoplarchus purpurescens*^C^	1: Elevated in wild fishes	High	Low	Low
	2: Wild-omnivore genes	High	Low	High
	3: Elevated in the lab genes	Low	High	High
	4: Wild-carnivore genes	High	High	Low
	5: Carnivore genes	Low	High	Low
	6: Omnivore genes	Low	Low	High
*Xiphister atropurpureus*^*O*^ and *Phytichthys chirus*^O^	1: Elevated in wild fish	High	Low	–
	2: Elevated in LC fish	Low	High	–

PSG: Positively Selected Gene when comparing sequences (PAML and Datamonkey) among wild-caught fishes of the four prickleback species

##### Liver

Overall, the most interesting finding for the liver with regards to transcriptomic analyses was how few genes showed changes in expression in the fishes fed the different diets. While 37 genes were differentially expressed between WF, LO and LC *X. mucosus*^H^, 30% of genes were annotated (Fig. 5). Cluster 1 (elevated in wild fish) consisted of genes for lipid metabolism (Table 6, Supplementary Table S7). Cluster 2 (wild-omnivore genes) consisted of genes for gluconeogenesis, while Cluster 5 (carnivore genes) contained genes responsible for the cellular processes associated with protein digestion and metabolism.

**Table 6:** Differentially Expressed Genes relevant to metabolism and digestion in all tissues for *Xiphister mucosus*^H^

Tissue	Cluster	Gene	Function	W fish	LC fish	LO fish
Liver	1	Apolipoprotein	Lipid metabolism	High (+ + + +)	Low (+ +)	Low (+ +)
	2	Phosphoenolpyruvate carboxykinase	Gluconeogenesis	High (+ + + + +)	Low ( +)	High (+ + + +)
Pyloric ceca	1	Fatty acid synthase	Fatty acid biosynthesis	High (+ + + + +)	Low ( +)	Low (+ + +)
	1	Endothelial lipase	Lipid metabolic process	High (+ + + + +)	Low ( +)	Low (+ + +)
	1	72 kDa type IV collagenase	Collagen catabolism	High (+ + + + +)	Low ( +)	Low (+ + +)
	1	Cathepsin B, an endopeptidase	Protease	High (+ + + + +)	Low ( +)	Low (+ + +)
	1	Lanosterol synthase	Cholesterol biosynthesis	High (+ + + +)	Low ( +)	Low (+ + + +)
	2	Fatty acid synthase	Fatty acid biosynthesis	High (+ + + +)	Low ( +)	High (+ + + +)
	2	Plectin	microtubules	High (+ + + +)	Low ( +)	High (+ + + +)
	2	Collagenase 3	Collagen catabolism	High (+ + + +)	Low ( +)	High (+ +)
	2	Gastrotropin	Bile acid metabolism/lipid transport	High (+ + + + +)	Low ( +)	High ( +)
	2	Lanosterol 14-alpha demethylase	Cholesterol biosynthesis	High (+ + + +)	Low ( +)	High (+ + + +)
	2	3-Hydroxy-3-methylglutaryl-coenzyme A reductase	Cholesterol biosynthesis	High (+ + + +)	Low ( +)	High (+ + + +)
Mid-intestine	1	Collagenase	Collagen catabolism	High (+ + + + +)	Low ( +)	Low ( +)
	1	Chitinase	Chitin metabolism	High (+ + + + +)	Low ( +)	Low ( +)
	1	Fatty acid synthase	Fatty acid biosynthesis	High (+ + + + +)	Low ( +)	Low ( +)
	2	Lanosterol synthase	Cholesterol biosynthesis	High (+ + + +)	Low ( +)	High (+ +)
	3	Carboxypeptidase	Protein metabolism	Low ( +)	High (+ + + +)	High (+ + + +)
	3	Carboxypeptidase A1	Protein metabolism	Low ( +)	High (+ + + +)	High (+ + + +)
	3	Carboxypeptidase B	Protein metabolism	Low ( +)	High (+ + + +)	High (+ + + +)
	3	Trypsin	Protein metabolism	Low ( +)	High (+ + + +)	High (+ + + +)
	3	Trypsin 3	Protein metabolism	Low ( +)	High (+ + + +)	High (+ + + +)
	3	Chymotrypsin-like elastase family member 1	Protein metabolism	Low ( +)	High (+ + + +)	High (+ + + +)
	3	Endoplasmic reticulum aminopeptidase 1	Protein metabolism	Low ( +)	High (+ + + +)	High (+ + + +)
	3	Mannosyl-oligosaccharide glucosidase	Glycolysis	Low ( +)	High (+ + + +)	High (+ + + +)
	3	Neuropeptidase Y receptor 2	Feeding behavior	Low ( +)	High (+ + + +)	High (+ + + +)

Gradient of expression is depicted by plus signs, in that one ( +) is low expression to a roughly fivefold increase (+ +  +  + +)

##### Pyloric ceca

For most species, there were more differentially expressed genes (DEGs) in the pyloric ceca than the liver. For instance, there were 183 DEGs when comparing WF, LO and LC *X. mucous*^H^, out of which 49.2% of genes were annotated (Fig. 5). Cluster 1 (elevated in wild fish) consisted of various genes involved in fatty acid and cholesterol biosynthesis, as well as in lipid, collagen, and protein metabolism (Table 6). Cluster 2 (wild-omnivore genes) consisted of various genes involved in fatty acid and cholesterol biosynthesis as well as collagen and bile acid metabolism.

##### Mid-intestine

There were consistently several hundred DEGs amongst the lab and wild-caught fishes when examining the mid intestine. There were 336 DEGs when comparing WF, LC, and LO *X. mucosus*^H^, out of which 37.8% were annotated (Fig. 5). Cluster 1 (elevated in wild fish) contained genes for collagen catabolism, chitin metabolism, and fatty acid biosynthesis (Table 6). Cluster 2 (wild-omnivore genes) contained genes for cholesterol biosynthesis. Cluster 3 (elevated in the lab genes) contained genes were involved in protein metabolism, glycolysis, and feeding behavior.

##### Comparisons among all tissues

**Fig. 6:**
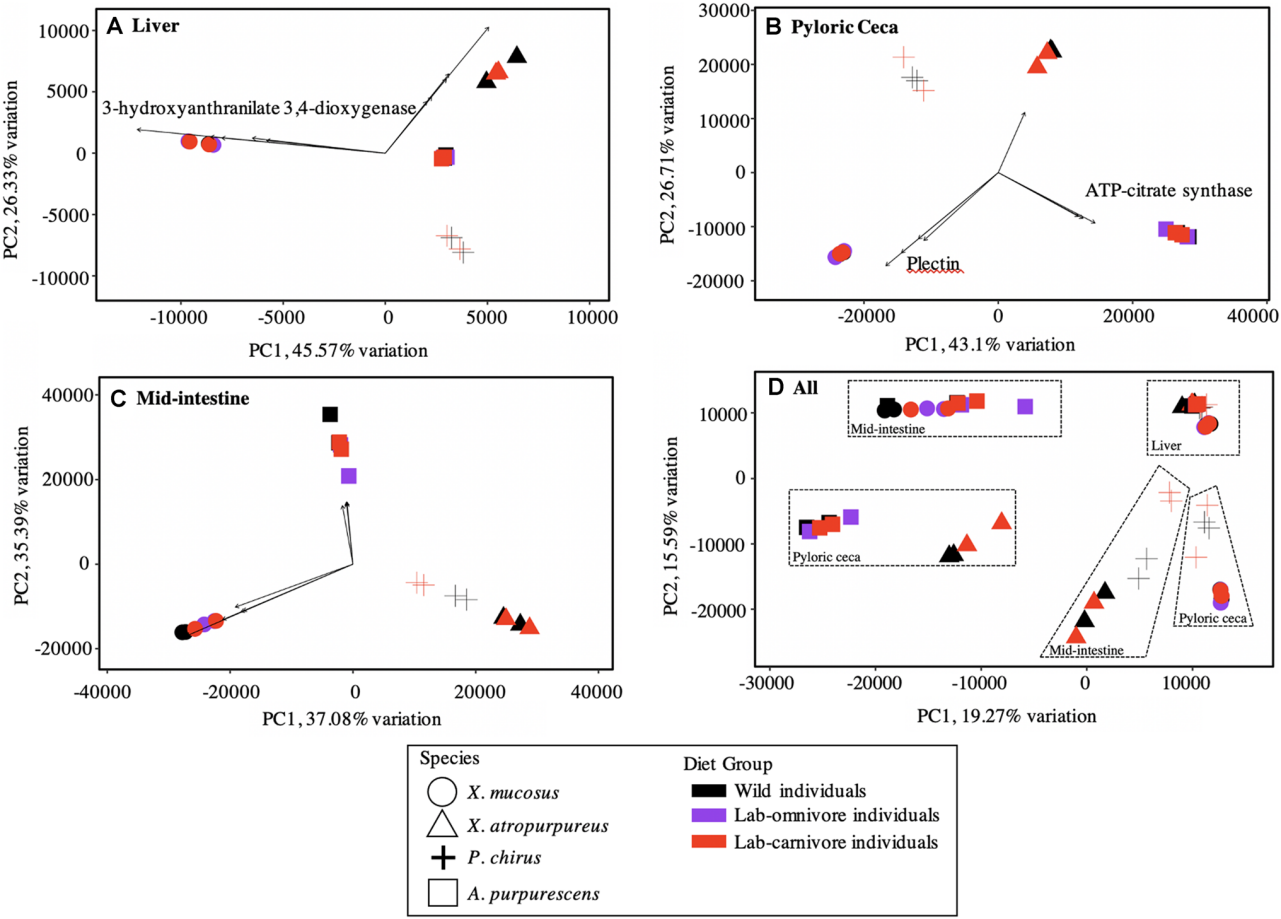
PCA plot of gene expression data from the four species and **A** liver, **B** pyloric ceca, **C** mid-intestine, and **D** all tissues combined with all species and diet groups. Shapes represent species. Spheres depict *X. mucosus*^H^, triangles depict *X. atropurpureus*^O^, plus sign ( +) depict *P. chirus*^O^, and squares depict *A. purpurescens*^C^. Colors represent diet, with wild individuals in black, lab-omnivore individuals in purple, and lab-carnivore individuals in red. Vectors in panels **A**–**C** indicate the ‘weight’ in different directions for the genes driving differences along each PC (fall within the top 5% of loadings range). The full gene list can be found in Supplementary Table S10 and the genes of interest that are related to digestion and metabolism are labeled on the graph

We generated principal components analysis (PCA) plots for each tissue based on the first two PCs, which explain most of the variation (Fig. 6). In the liver, pyloric ceca, and mid-intestine, individuals group by species and within each species, by diet group. In the liver, there is less variation than the digestive tissues as shown by the relatively more constrained axes, and 3-hydroxyxyanthranilate 3,4-dioxygenase explains some of the variation in *X. mucosus*^H^. In the pyloric ceca, the gene for plectin explains some of what sets *X. mucosus*^H^ apart from the other species, while ATP-citrate synthase explains some of the variation in *A. purpurescens*^C^ (Fig. 6B, Supplementary Table S10). When all tissues are combined, we find grouping by tissue, with the liver being the least plastic and the mid-intestine exhibiting the most variation and plasticity followed by the pyloric ceca.

In addition, we generated a correlation matrix of the gene expression patterns of all the tissues we measured. The samples tended to cluster by tissue, species, and diet (WF, LO, LC), with the exception of liver, which did cluster by tissue and species, but not by diet, reflecting the lack of expression changes seen in the lab fishes vs the wild fishes (Supplemental Fig. S24). *P. chirus*^O^ showed the most divergent expression patterns, particularly in their livers (distinct cluster 1), where they did not group with any other species (Supplemental Fig. S24). Interestingly, *A. purpurescens*^C^ and *X. atropurpureus*^O^ group adjacent to each other for pyloric ceca, and liver, but *X. atropurpureus*^O^ grouped more with *P. chirus*^O^ for mid intestine gene expression patterns (Supplemental Fig. S24). *X. mucosus*^H^ tended to cluster more with itself, representing its unique status as the herbivorous fish in this study. These data support what is shown in the PCA plots (Fig. 6).

## Discussion

This study showed that prickleback fishes have variable responses to dietary perturbations, thus indicating that natural diet and species identity affect digestion and metabolism in some predictable (Tables 1, 2) and unpredictable ways. Gut length largely varied with diet quality (Fig. 2), agreeing with our expectations that herbivorous fishes generally have longer guts than carnivorous fishes. Moreover, although we did see differences in expressed genes in response to dietary shifts, the liver appears to be less flexible than other tissues, hinting that liver metabolism may be where dietary specialization manifests the most in prickleback fishes, and in a species-specific manner. We will address study objectives one and two in order.

### Objective 1: comparisons of wild-caught fishes

One of the biggest determinants of gut length is intake: animals eating lower-quality foods (higher fiber, less soluble nutrients) have higher intake, and hence, more rapid transit of digesta through their guts (German 2011). This rapid transit of digesta requires a longer gut to allow for adequate nutrient absorption in terms of time and surface area (Al-Hussaini 1947; German and Horn 2006; Kapoor et al. 1975; Horn 1989; Kramer 1995; Leigh et al. 2018; Davis et al. 2013). Indeed, wild herbivorous *X. mucosus*^H^ have longer guts than the other prickleback species (German and Horn 2006; German et al. 2014), and a longer gut often equates to greater absorptive surface area achieved through increased intestinal folding and more microvilli (Karasov and Hume 1997; Starck 2005; Secor 2008; German et al. 2010; Leigh et al. 2018). Coinciding with these longer guts, tubulin alpha chain, which is a constituent of microtubules that are critical in microvilli structure in the gut (Paradela et al. 2005), is under positive selection in the pyloric ceca of *X. mucosus*^H^ (Supplemental Table S4), and this species shows more positive gut length allometry as they grow than the other prickleback species we studied (German et al. 2014). The PCA vectors also show that plectin genes make *X. mucosus*^H^ stand apart from the other taxa in terms of genes expressed in the pyloric ceca (Fig. 6, Supplementary Table S10). Plectins are also involved with microvilli lengthening and structure (Wiche 1998; Weisz and Rodriguez-Boulan 2009). Hence, there is clear evidence that the herbivorous *X. mucosus*^H^ not only displays a longer gut, but has genes under selection that contribute to greater epithelial surface area, all of which allow them to thrive on an algal diet. The pyloric ceca and mid-intestine are similar in function, as both are highly absorptive (Buddington and Diamond 1987; Heras et al. 2020), and therefore, finding genes that express proteins involved with increasing surface area in the pyloric ceca agrees with the function of that tissue.

To our knowledge, only one other study (Saba 2004) examined how diet affects the δ^13^C and δ^15^N signatures of prickleback fishes. Examining the isotopic signature of tissues with a high protein turnover rate and that are metabolically active, such as the liver, allows us to track the isotopic composition of diet closely (Karasov and Martínez del Rio 2007). In wild fishes (Supplemental Figure S13), we isotopically confirmed that the sister taxa *X. mucosus*^H^ and *X. atropurpureus*^O^ consume different diets, with *X. atropurpureus*^O^ being enriched from the carbon and nitrogen perspective. As noted previously, WF *P. chirus*^O^ are more enriched from the carbon perspective compared to WF *X. mucosus*^H^ and *P. chirus*^O^ consumes the most crustaceans among the four studied species (German et al. 2015). Contrary to expectations (e.g., Barton 1982; German et al. 2004; German and Horn 2006; German et al. 2014), the stable isotopic signature of *A. purpurescens*^C^ suggests a mixed diet that should be examined in more detail, especially since they do not appear to assimilate algal protein in the lab (Fig. 3). *Anoplarchus purpurescens*^C^ consume more worms in nature than the other pricklebacks, which may skew their δ^15^N signatures to be lower than the other fishes consuming more crustaceans (Saba 2004; German and Horn 2006), although the elevated expression of *α*-mannosidase in the mid-intestine of *A. purpurescens*^C^ (Supplemental Table S9) may be involved with digestion of glycoproteins in crustaceans (Kuballa and Elizur 2008).

The genes under positive selection among the prickleback species are mostly relating to lipid metabolism in the liver (Table 4; Fig. 4), and mostly in *P. chirus*^O^, although niacin metabolism is also identified as an important gene in *X. mucosus*^H^ using the PCA vectors (Fig. 6). Fishes consuming higher carbohydrate diets seem to require more niacin, and herbivorous diets are lower in tryptophan (a niacin precursor) and niacin itself than carnivorous diets (Shiau and Suen 1992; Hansen et al. 2015). Because they naturally have a higher-carbohydrate diet in comparison to the other studied fishes, *X. mucosus*^H^ may have ramped up niacin synthesis pathways (specifically 3-hydroxyanthranilate 3,4-dioxygenase) to ensure they have enough NAD and NADPH for metabolism like the Citric Acid Cycle and lipid synthesis. With the strong interest in moving towards more sustainable, plant-based aquaculture feeds, in addition to examining how herbivorous fishes digest algal diets (e.g., Heras et al. 2020), understanding what metabolic pathways are ramped up in herbivores is also important, and these findings on niacin metabolism in *X. mucosus*^H^ may provide a new avenue to explore in herbivorous fish aquaculture (e.g., Hansen et al. 2015). Several proteolytic genes were under positive selection in the pyloric ceca and mid intestine of the fishes (Supplemental Tables S3 and S4; Supplemental Figure S14), but interestingly, they were not all in the carnivores, as *X. mucosus*^H^ showed selection on elastase and trypsin. Although it is common to see elevated carbohydrase gene expression and enzyme activities in the guts of fishes eating more plant material (e.g., German et al. 2015, 2016; Heras et al. 2020), all fishes need protein, so it is not surprising to see selection on proteases in fishes consuming lower-protein diets, like *X. mucosus*^H^ (Heras et al. 2020).

### Objective 2: dietary flexibility in the laboratory

The flexibility of the prickleback gut length in response to the laboratory diets changed in accordance with intake, with LC fishes having shorter guts than LO fishes (Table 2, Fig. 2). Interestingly, changes in gut length are reflected on the molecular level, with the upregulation of genes involved in the generation of microtubules and muscle fiber in wild and LO fishes in comparison to LC fishes, particularly in *X. mucosus*^H^ (Table 6). This indicates changes on the molecular level to achieve a larger gut (Castoe et al. 2011). The changes in gut size and concomitant changes in gene expression support models of cellular hypertrophy (Starck 2005; Leigh et al. 2018) or cellular proliferation (Riddle et al. 2020) in fishes, that can affect gut surface area (e.g., Leigh et al. 2018).

The mid-intestine is the primary site of digestion of carbohydrates, fats, and proteins and it is a highly absorptive region of end products, vitamins, and minerals (Stevens and Hume 1995; Heras et al. 2020). As expected, we do see diet-dependent changes in gene expression profiles of the mid-intestine. When examining the genes showing differential expression, it is clear that *X. mucosus*^H^ (and to a lesser extent, *A. purpurescens*^C^) increased expression of proteolytic enzymes in response to the high-protein laboratory diets (Table 6). Serine proteases (including trypsin and chymotrypsin) were increased in expression on the LO and LC diets, concurring with increases in tryptic activity in these same species raised on high-protein diets (German et al. 2004), and showing the flexibility of the gut in response to the laboratory diets. Why the two natural omnivores, *X. atropurpureus*^O^ and *P. chirus*^O^, did not show many increases in gene expression for proteolytic enzymes or amino acid transporters in response to the LC diet is unclear, but the mid intestines of these taxa were more similar to each other than any of the other species (Fig. 6; Supplemental Fig. S24), suggesting some shared function among them. Finally, trypsin, carboxypeptidase, chitinase, and lipase expression in the mid intestine confirms the broader distribution of pancreatic cells along the stichaeid intestine (Heras et al. 2020), showing it is not confined to the pyloric cecal region, as proposed previously (Gawlicka and Horn 2006).

The pyloric ceca of fish plays a key role in digestion and absorption, functioning in enzymatic digestion and nutrient absorption, including lipid digestion (Williams 2019; Buddington and Diamond 1987; Stevens and Hume 1995). Similar to our results, the pyloric ceca transcriptome of salmon fingerlings fed varying oil (Jin et al. 2018) or carbohydrate (Betancor et al. 2018) sources upregulated genes involved in lipid metabolism. In prickleback fishes, we found that wild fishes upregulate genes involved in protein, carbohydrate, and lipid metabolism and converting nutrients to energy storage in the pyloric ceca, yet we do not see this pattern in fishes fed the LO and LC diets. Although we changed the carbohydrate content in both lab-formulated diets (Table 3), we do not see fishes fed laboratory-formulated diets respond to the differences in carbohydrate content in the pyloric ceca (German et al. 2004; Kim et al. 2014). Instead, genes involved in key carbohydrate metabolism pathways, such as glycolysis, gluconeogenesis, and pentose phosphate pathway, which is a major pathway for glucose breakdown in fish, were upregulated in the pyloric ceca of wild fishes. Interestingly, chitinase expression is upregulated in the pyloric ceca of wild *X. atropurpureus*^O^, and this taxon has moderate chitin digestive capability (German et al. 2015). Elevated ability to digest chitin and protein may be why, for the pyloric ceca, *X. atropurpureus*^O^ showed more similarity with *A. purpurescens*^C^ than other species when comparing the transcriptomics of all tissues (Fig. 6; Supplemental Fig. S24).

The pyloric ceca of *P. chirus*^O^ showed relatively few DEGs, and only wild individuals of this species upregulate genes to break down protein, even though we see many changes in the liver transcriptome and liver stable isotope signatures in *P. chirus*^O^. Genes for protein and fatty acid metabolism are under positive selection in wild fishes as well. For instance, consistent with their high protein natural diets, serine protease 27 is upregulated and under positive selection in *P. chirus*^O^ and *A. purpurescens*^C^ (Supplemental Table S8, Supplemental Fig. S14).

Although metabolic rate didn’t vary among the prickleback species fed different diets (see Supplemental Discussion for more information on metabolic rate), *X. mucosus*^H^ and *A. purpurescens*^C^ consuming the high-protein LC diet grew fastest in the laboratory, as was expected (Fris and Horn 1993; Horn et al. 1995; Leigh et al. 2018). Thus, each of the prickleback species tolerated the carnivorous diet well.

The liver stable isotopic data (Fig. 3) showed that fishes assimilated and metabolized the formulated diets, agreeing with our expectations that fishes fed either an omnivore or carnivore diet in the lab would have stable isotopic signatures reflecting the laboratory diets. Given that fish liver tissue can isotopically turn over within a 28 day time frame (i.e., the length of our feeding trial; Guelinckx et al. 2007; German and Miles 2010; Matley et al. 2016), the measured isotopic signatures likely reflect an equilibrium value for tissue-diet discrimination (German and Miles 2010). The fishes largely showed typical tissue-diet discrimination for δ^13^C and δ^15^N. If the species were all the same in how they digested and metabolized the diets, they would completely overlap in the laboratory in terms of their δ^13^C and δ^15^N signatures (Saba 2004), but this was not the case. For one, *A. purpurescens*^C^ did not appear to assimilate much of the algal nitrogen in the omnivorous diet, as their signatures were nearly identical on the two laboratory diets, suggesting this species was primarily digesting the fish protein within the LC and LO diets. Although not statistically significant, *X. mucosus*^H^ did show some variation in δ^15^N on the two diets, with fish fed the LC diet trending upwards, suggesting that this species was indeed assimilating at least some algal protein on the LO diet, as would be expected for herbivorous pricklebacks (Horn et al. 1986a, b; Fris and Horn 1993). The δ^15^N of LO *X. mucosus*^H^ was also significantly lower than in LO *A. purpurescens*^C^, suggesting these two species differed in how they digested and metabolized the LO diet. *P. chirus*^O^ stood out both in how much their isotopic signature and liver transcriptome changed when being fed the carnivore diet in the laboratory. These two factors may be related and are discussed below.

#### Liver exhibits species-specific responses

Although there were hundreds of genes differentially expressed among the three diet groups in the intestine (pyloric ceca and mid-intestine), three of the four species did not appreciably alter gene expression in the liver in response to different diets in the laboratory, and the liver showed fewer shared orthologs among wild-caught fishes than the other tissues. Consistent with previous studies that compared the liver with the intestine, there were few changes in the gene expression of the liver of prickleback fishes fed different diets, and the most responsive pathway is lipid metabolism in the laboratory-fed fishes (De Santis et al. 2015a). In our PCA plots (Fig. 6) and correlation matrix (Supplemental Fig. S24), it is clear that the liver shows the most species-specific expression patterns, and changed the least in response to the LO and LC diets. In the PCA plots, the liver axes were the smallest (i.e., covers the least amount of variable space), and the liver was in a tight space within the plot including all tissues and treatments (Fig. 6D). These liver expression patterns agree with previous investigations showing more species and population-level liver gene expression patterns (Bernal et al. 2019; Merkin et al. 2012; Betancor et al. 2018).

Wild fishes of *X. mucosus*^H^*, X. atropurpureus*^O^ and *A. purpurescens*^C^ showed elevated expression of genes for lipid metabolism and glucose metabolism in their livers, yet these same pathways were downregulated on the laboratory diets, similar to Atlantic Salmon fed plant-based diets in the laboratory (Król et al. 2016; De Santis et al. 2015a). The relatively few changes in liver gene expression in fishes fed formulated diets in the laboratory suggests that gene expression of the liver is not readily altered in the face of dietary perturbations, and instead, is more specialized by species and likely reflects natural diet. Merkin et al. (2012) also showed the liver is specialized based on species identity, when comparing liver gene expression profiles with other tissues in vertebrate animals. Similarly, Atlantic salmon fed high or low starch diets revealed population-level, not dietary, effects on liver metabolic pathway regulation (Betancor et al. 2018), whereas this same species showed few liver DEGs in response to dietary variation, unlike their pyloric ceca, stomach, or distal intestine, which showed increased expression of genes involved in lipid metabolism (Jin et al. 2018). Overall, only a handful of studies examine changes in liver gene expression in response to dietary differences, and several of them find that the liver exhibits a more tissue-specific response when comparing different diets and tissues, and we find that the prickleback livers’ response is also species-specific and more tuned to natural diet. This is a truly novel result in this comparison of closely related species and argues that the liver may be an important aspect of dietary specialization.

While there are not many metabolic genes under positive selection in the liver, glucose-6-phosphate 1-dehydrogenase (G6PD), the enzyme involved in the first step of the pentose phosphate pathway, and endothelial lipase, an enzyme that breaks down plasma lipids for entry into cells, are under positive selection in *P. chirus*^O^ (Fig. 4). Finding G6PD under positive selection in the liver agrees with previous studies that have found lipid metabolism pathways in the liver to be responsive to nutritional stress (De Santis et al. 2015a). Interestingly and contrary to what is found in the other three species, *P. chirus*^O^ shows large changes in gene expression in the liver when comparing wild fishes to fishes fed a carnivore diet in laboratory (Supplemental Figure S16). *P. chirus*^O^ on the LC diet upregulated genes involved in lipid metabolism, fatty acid synthesis, and bile acid biosynthesis, indicating this species ability to metabolize the LC diet. Further, the stable isotope signature of LC *P. chirus*^O^ fish livers were significantly more enriched from the carbon perspective compared to the other species (Fig. 3). While a typical tissue-diet discrimination factor for ^13^C (∆^13^C) in the liver ranges  ±1.5‰ and most of the species studied here follow this expectation (Karasov and Martínez del Rio 2007; Caut et al. 2009), LC *P. chirus*^O^ fish livers show a ∆^13^C of + 4‰. Additionally, when comparing only wild fishes, wild *P. chirus*^O^ only significantly differ in ^13^C signature from wild *X. mucosus*^H^ fish, showing that they are not dramatically different from the other species in the wild (Supplemental Figure S13). It is worth noting that we did not extract lipids from the liver before stable isotope analysis. Lipids are typically depleted in δ^13^C relative to their dietary source (Post et al. 2007), and in this study we found enrichment in δ13C in *P. chirus*^O^, making the enrichment unlikely to do with liver lipid. We did try adjusting our data for lipid content with the equations in Post et al. (2007), which did not appreciably change anything. Instead, there may be a metabolic explanation for the large ∆^13^C in *P. chirus*^O^. Rito et al. (2019) found that an excess of ^13^C in seabass could be explained by variability in pentose phosphate pathway activity (Jin et al. 2014; Rito et al. 2019). It is possible that variability in the pentose phosphate pathway could explain the high ∆^13^C in *P. chirus*^O^, especially because G6PD, the enzyme involved in the first step of the pentose phosphate pathway, is highly expressed in LC *P. chirus*^O^ (Supplemental Table S7, Supplemental Figure S16) and is under positive selection in wild *P. chirus* (Fig. 4). G6PD has previously been identified as influenced by diet and population in a feeding experiment on Atlantic Salmon (Betancor et al. 2018). Alternatively, a different aspect of their metabolism in the liver can selectively be routing in more ^13^C to the liver, leading to an enriched signal in relation to the other fishes (Karasov and Martínez del Rio 2007), but still unique to this species.

### Conclusions

In this study, we sought to understand dietary specialization in closely related, sympatric, prickleback fishes with different diets. We confirmed that gut length varies with diet quality, even intra-specifically, but there are limits to this since the herbivorous *X. mucosus*^H^ always had a longer gut than the other species, even when they consumed the same diet in the laboratory. Thus, gut length definitely has a genetic underpinning and is not just plastic (German and Horn 2006; Sullam et al. 2015; Riddle et al. 2020). We observed positive selection on, and increased expression of, genes that would contribute to increased epithelial surface area in *X. mucosus*^H^, but not the other taxa. Our transcriptomics data confirm the plasticity of the mid intestine in pricklebacks, affirming plasticity of digestive enzyme activities observed previously (German et al. 2004; Gawlicka and Horn 2005, Gawlicka and Horn 2006; Kim et al. 2014). Therefore, the gut itself can display enough plasticity to allow even an herbivore to digest a carnivorous diet, but the opposite is not true, as carnivorous fishes do not tolerate herbivorous diets (Król et al. 2016), and *A. purpurescens*^C^ did not appear to assimilate algal protein in this study. However, the real novel finding is how inflexible the liver gene expression patterns are. In fishes, true specializations may manifest with food acquisition (i.e., mouth morphology and biomechanics; Burress et al. 2020) and then on the nutrient processing side of things once nutrients are absorbed into the blood stream (Wilmott et al. 2005; DeSantis et al. 2015a; Betancor et al. 2018). If the liver is inflexible, this means that although the gut itself can respond to shifts in nutrient concentrations entering the gut, the liver may not be equipped to process excesses of a nutrient class (e.g., glucose in carnivores, or amino acids in herbivores; Shiau and Suen 1992; Ferrais and Diamond 1997). Indeed, this may be related to the variance in signals received in the different tissues: the gut deals with large swings in nutrient concentrations, whereas the liver encounters much smaller changes in concentrations in the blood stream (Ferrais and Diamond 1997). Moreover, given the differences in niacin synthesis pathways we observed among the fishes, vitamin requirements may also be inflexible, and certainly vary among species based on natural diets (Shiau and Suen 1992; Hansen et al. 2015). Therefore, by taking more of a systems approach, we identify areas on which to focus in the quest to understand dietary specializations in fishes in ecological, evolutionary, and aquaculture contexts. Much work is still necessary to elucidate the mechanisms underlying liver specialization, which results in the liver being less plastic than digestive tissues in response to different diets. For instance, it is the loss of the uricase gene in primates that sets their liver function, particularly in response to fructose metabolism, apart from other mammals (Kratzer et al. 2014). Similar patterns, with different genes (e.g., G6PD), may emerge in fishes, and this study should help generate hypotheses for new directions in dietary specialization research. We do recognize that our low sample sizes for the transcriptomics analysis (*n* = 2) may limit what we observed in this study, both, in terms of genes under selection (objective one), and for differentially expressed genes in response to a dietary perturbation (objective two), and thus, we need to sample more individuals in future studies. Nevertheless, the dataset presented here provides new directions in the field of fish nutritional physiology.

## Supplementary Information

Below is the link to the electronic supplementary material.

Supplementary file1 (PDF 4400 KB)

## Data Availability

The datasets presented in this study can be found in online repositories, accession number for the Bioproject is PRJNA738880, where the Biosamples and SRA files are located. All other data can be found here: https://german.bio.uci.edu/Supplements.html.
