# Supplementary material for: Comparative transcriptomics reveal tissue level specialization towards diet in prickleback fishes
Source: J Comp Physiol B. 2022 Jan 25;192(2):275–95. doi: 10.1007/s00360-021-01426-1 (PMC8894155; doi:10.1007/s00360-021-01426-1)
Supplement: Supplementary file 1 — Supplementary file1 (PDF 4400 KB) [file 360_2021_1426_MOESM1_ESM.pdf]

Comparative transcriptomics reveal tissue level specialization towards diet in prickleback fishes

Michelle J. Herrera[1], Joseph Heras[2], Donovan P. German[1]

[1]Department of Ecology and Evolutionary Biology, University of California, Irvine, 321 Steinhaus Hall, Irvine, Ca 92697-2525, USA

[2]Department of Biology, California State University, San Bernardino, 5500 University Parkway, San Bernardino, CA 92407, USA

Corresponding author

Michelle J. Herrera mjherre01@gmail.com

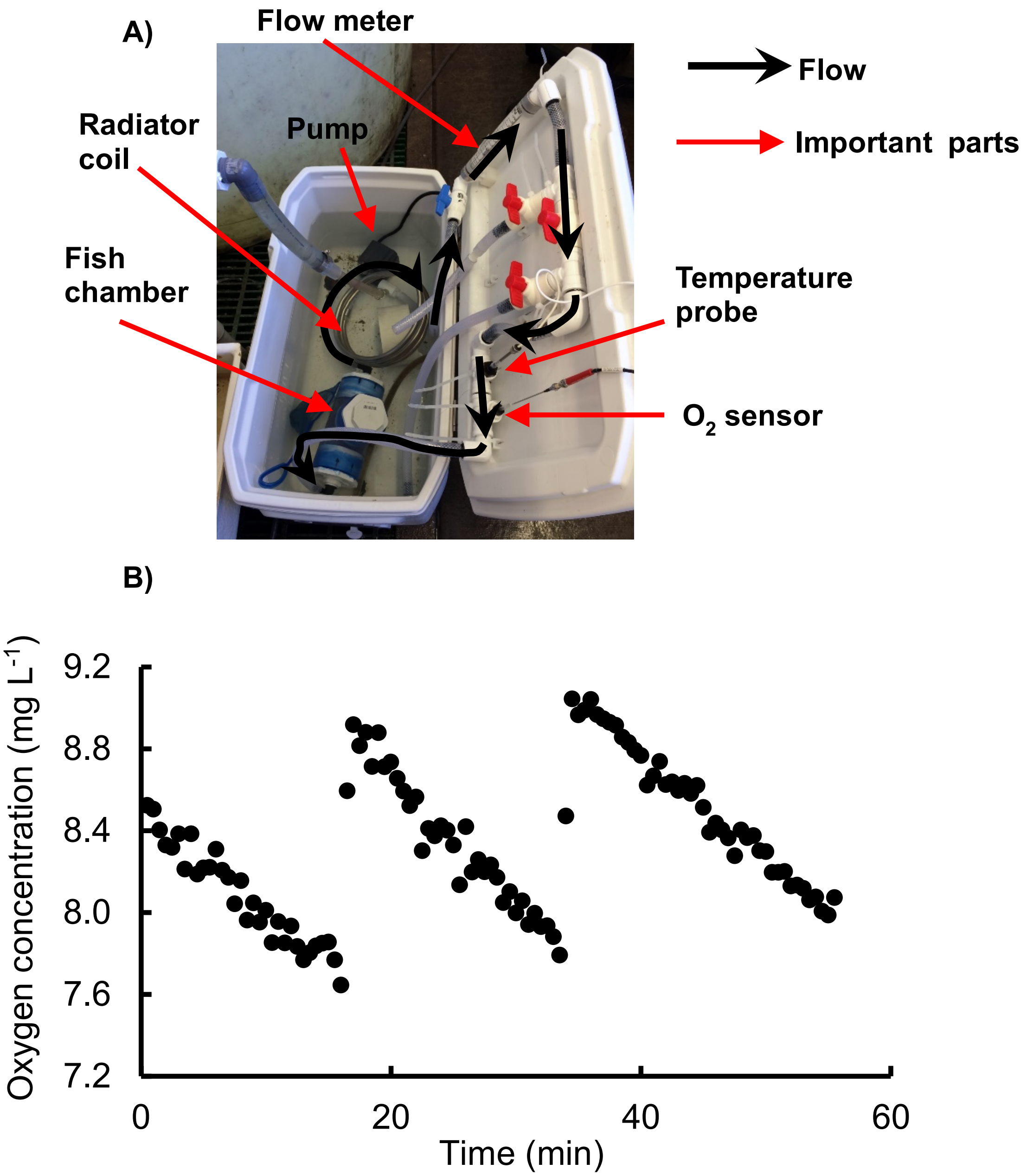

Supplemental Figure S1. A. Respirometer setup showing the chamber submerged in ambient seawater in the closed, recirculating configuration with thermistor and oxygen probe in series. The oxygen consumption of each fish was measured individually in 15-minute intervals after a 30 min acclimation period to the respirometry chamber. Following each 15-min interval, the valves of the system were opened manually to the open configuration to exchange with the flow-through, ambient seawater before being closed again for the next measurement period. B. Representative plot showing the oxygen concentrations in the respirometer over time during measurements of fish metabolic rate. The portions with the negative slopes are the measurement periods when the system was closed (15 min intervals). The system was then opened again for several minutes to be flushed by new seawater from the flow-through system, and then closed again to take another measurement on the same fish. This process was repeated three times. The above traces are from an individual of *Anoplarchus purpurescens*[C] that weighed 6.65g.

X. mucosus^{H} wild individuals

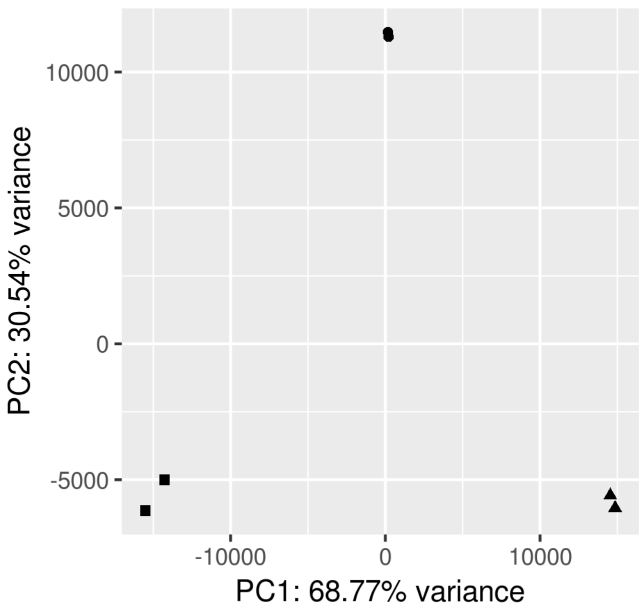

Supplemental Figure S2: *X. mucosus*^{H} wild fish replicates. PCA plot to depict the quality check analysis of individual replicates within the same species and diet group and across the three tissues we sequenced for transcriptomic data. Spheres depict Liver replicates, Squares depict pyloric ceca replicates, and triangles depict mid-intestine replicates.

X. mucosus[H] lab omnivore individuals

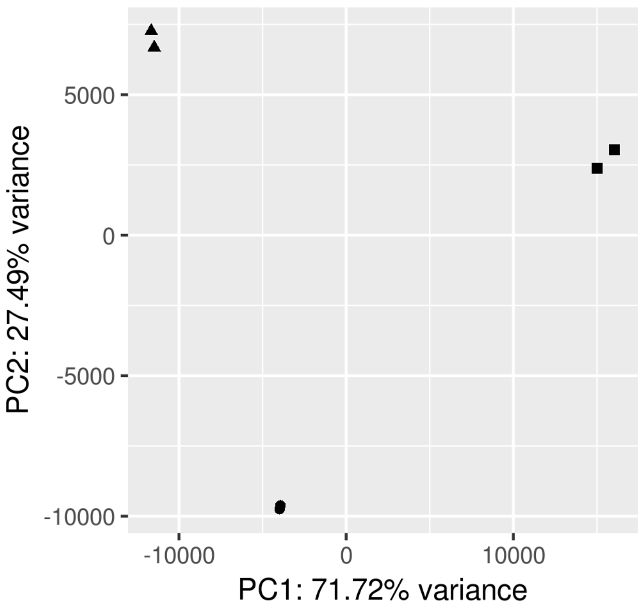

Supplemental Figure S3: *X. mucosus*[H] laboratory omnivore diet fish replicates. PCA plot to depict the quality check analysis of individual replicates within the same species and diet group and across the three tissues we sequenced for transcriptomic data. Spheres depict Liver replicates, Squares depict pyloric ceca replicates, and triangles depict mid-intestine replicates.

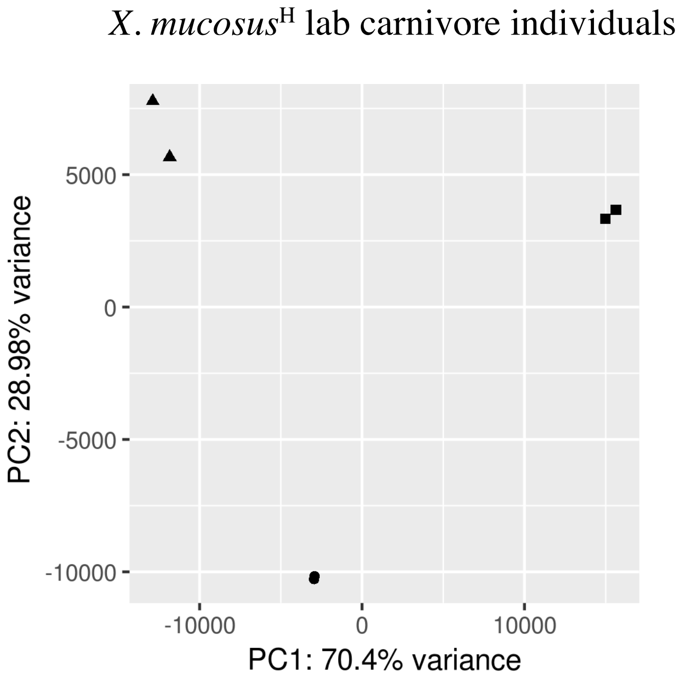

Supplemental Figure S4: *X. mucosus*^{H} laboratory carnivore diet replicates. PCA plot to depict the quality check analysis of individual replicates within the same species and diet group and across the three tissues we sequenced for transcriptomic data. Spheres depict Liver replicates, Squares depict pyloric ceca replicates, and triangles depict mid-intestine replicates.

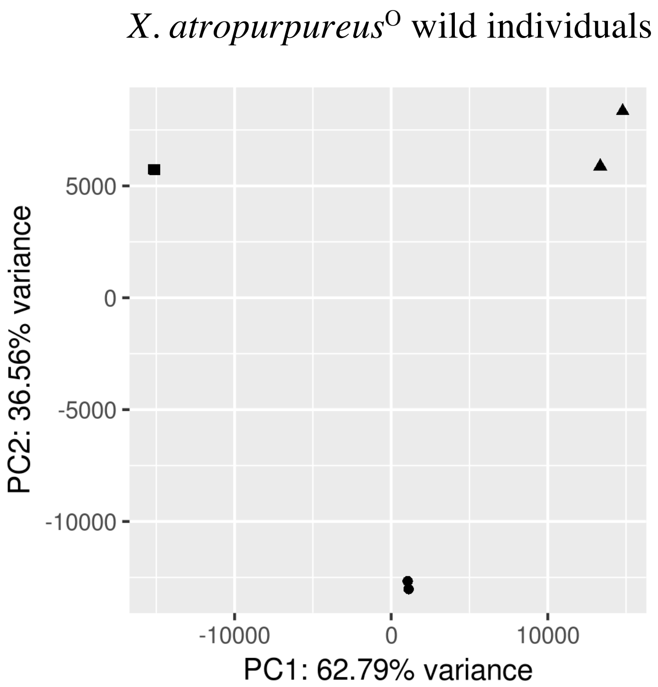

Supplemental Figure S5: *X. atropurpureus*^{O} wild fish replicates. PCA plot to depict the quality check analysis of individual replicates within the same species and diet group and across the three tissues we sequenced for transcriptomic data. Spheres depict Liver replicates, Squares depict pyloric ceca replicates, and triangles depict mid-intestine replicates.

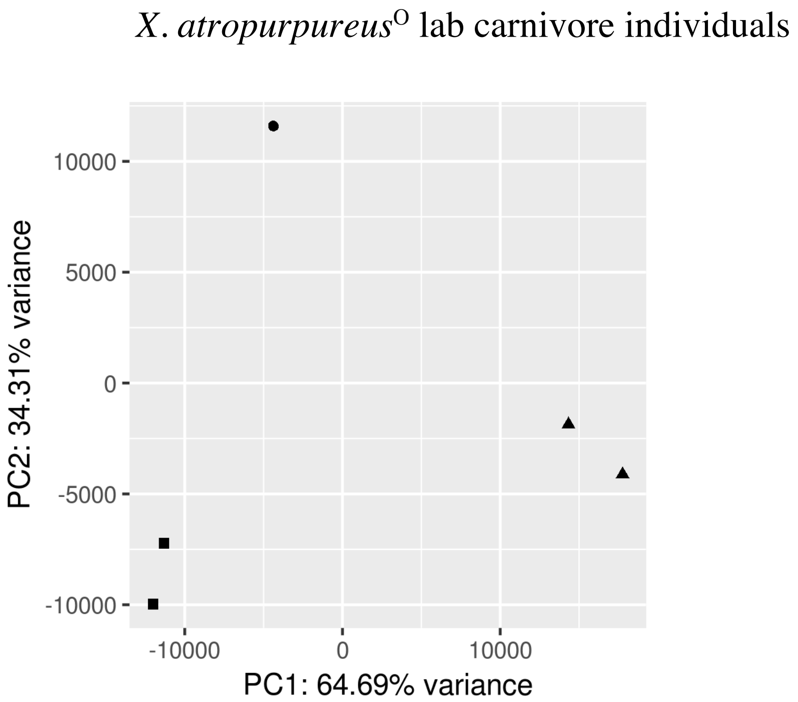

Supplemental Figure S6: *X. atropurpureus*[O] laboratory carnivore diet replicates. PCA plot to depict the quality check analysis of individual replicates within the same species and diet group and across the three tissues we sequenced for transcriptomic data. Spheres depict Liver replicates, Squares depict pyloric ceca replicates, and triangles depict mid-intestine replicates.

P. chirus[O] wild individuals

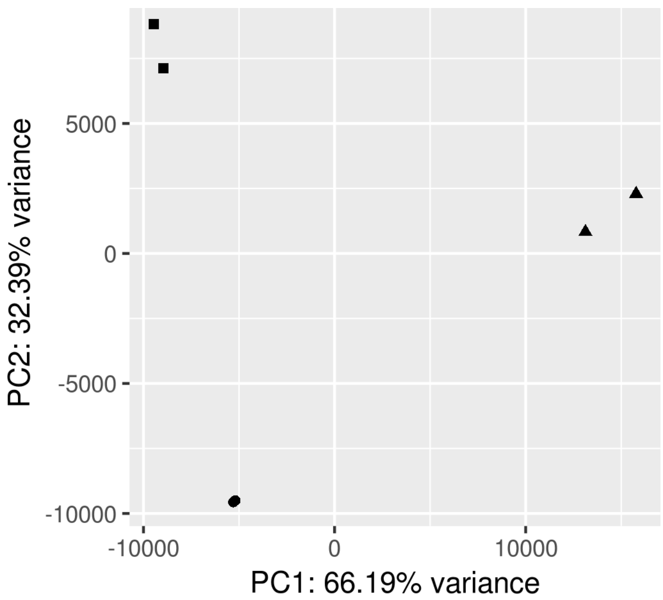

Supplemental Figure S7: *P. chirus*[O] wild fish replicates. PCA plot to depict the quality check analysis of individual replicates within the same species and diet group and across the three tissues we sequenced for transcriptomic data. Spheres depict Liver replicates, Squares depict pyloric ceca replicates, and triangles depict mid-intestine replicates.

P. chirus[O] lab carnivore individuals

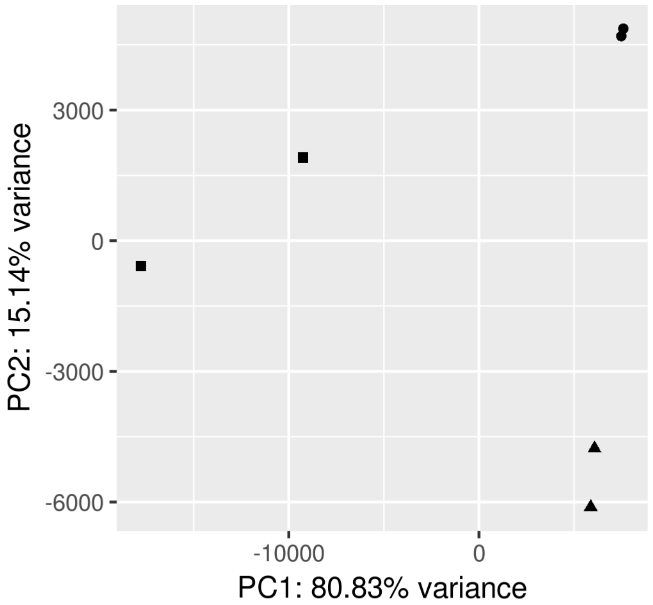

Supplemental Figure S8: *P. chirus*[O] laboratory carnivore diet replicates. PCA plot to depict the quality check analysis of individual replicates within the same species and diet group and across the three tissues we sequenced for transcriptomic data. Spheres depict Liver replicates, Squares depict pyloric ceca replicates, and triangles depict mid-intestine replicates.

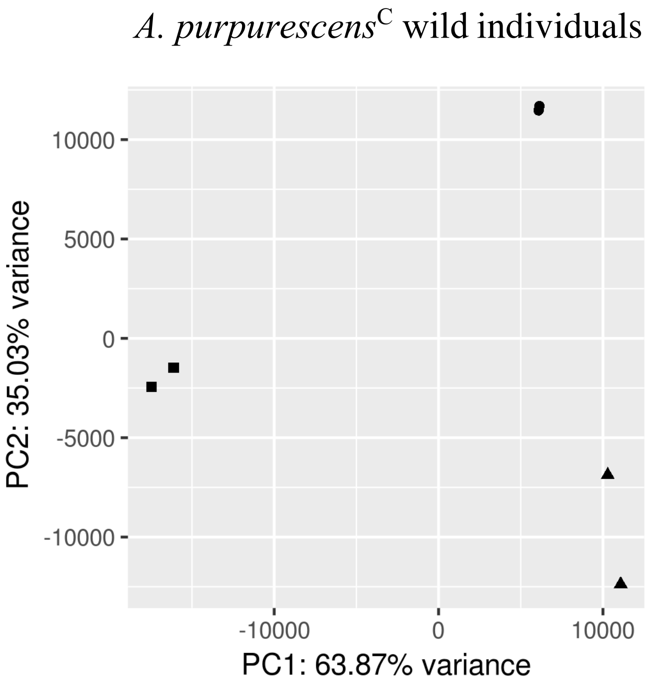

Supplemental Figure S9: *A. purpurescens*^{C} wild fish replicates. PCA plot to depict the quality check analysis of individual replicates within the same species and diet group and across the three tissues we sequenced for transcriptomic data. Spheres depict Liver replicates, Squares depict pyloric ceca replicates, and triangles depict mid-intestine replicates.

A. purpurescens^C lab omnivore individuals

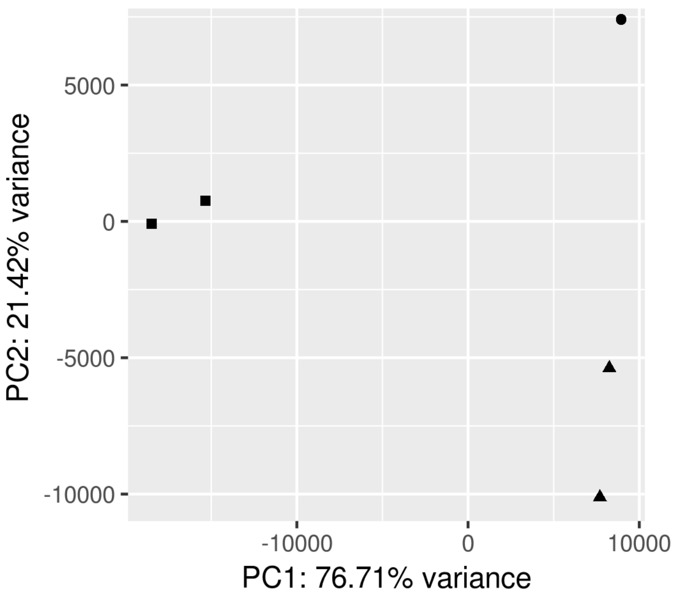

Supplemental Figure S10: *A. purpurescens*^{C} laboratory omnivore diet replicates. PCA plot to depict the quality check analysis of individual replicates within the same species and diet group and across the three tissues we sequenced for transcriptomic data. Spheres depict Liver replicates, Squares depict pyloric ceca replicates, and triangles depict mid-intestine replicates.

A. purpurescens[C] lab carnivore individuals

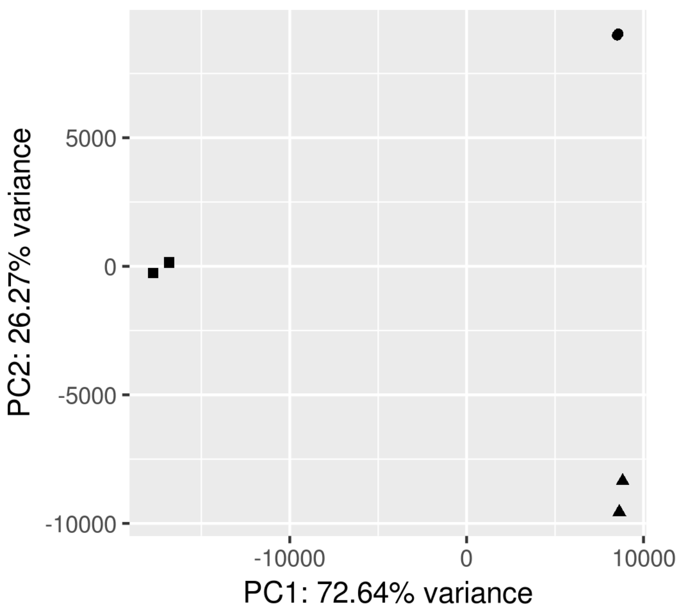

Supplemental Figure S11: *A. purpurescens*[C] laboratory carnivore diet replicates. PCA plot to depict the quality check analysis of individual replicates within the same species and diet group and across the three tissues we sequenced. Spheres depict Liver replicates, Squares depict pyloric ceca replicates, and triangles depict mid-intestine replicates.

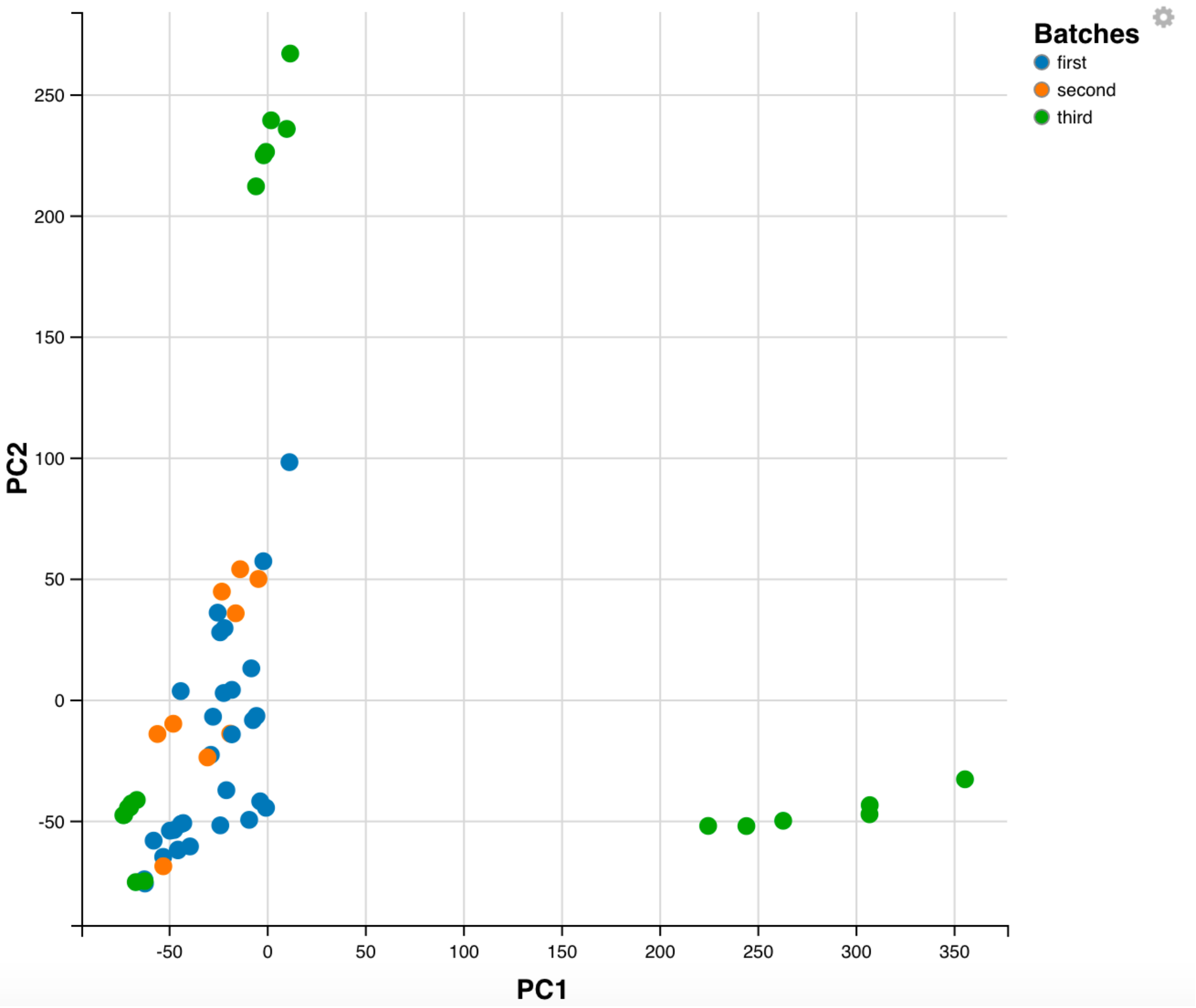

Supplemental Figure S12: PCA plot generated with Batch Quality Check Results. Color indicated batch (blue is first run, orange is second run, green is third run. Samples do not cluster by batch. The standardized Pearson correlation coefficient is 0.87 and the Cramer's V is 0.7, indicating batch does not fully interfere with the signal, with batch 3 showing the most uniqueness because this batch contained the liver samples. All species are represented in each cluster of liver samples (batch 3) on the plot, showing that they are dispersed throughout and not grouping by sample.

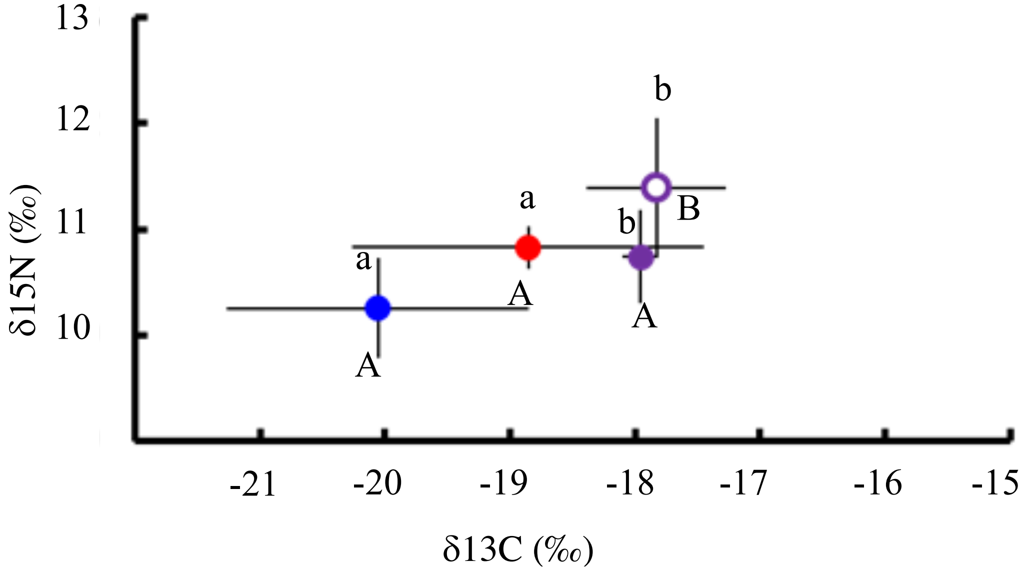

Supplemental Figure S13. Carbon and nitrogen (‰) dual isotope plot of wild-caught prickleback fishes. *X. mucosus*[H] (blue sphere), *X. atropurpureus*[O] (purple ring), *P. chirus*[O] (purple sphere), and *A. purpurescens*[C] (red sphere). Values are mean ± standard deviation. Interspecific comparisons were made with ANOVA. Significant differences ($P<0.05$) for δ15N indicated with capital letters, whereas lower case letters indicate significant differences in δ13C values. Symbols sharing a capital or lower case letter are not significantly different.

**Supplemental Table S1** Stable Isotope ANOVA and Tukey results within each species across diet groups. And wild fish, lab-omnivore fish, and lab-carnivore fish ANOVA and Tukey results				
Species		ANOVA	Tukeys HSD	
X. mucosus[H]	Carbon	P=0.073	Wild-LC	p=0.0808
		$F_{2,7}$=3.893	Wild-LO	p=0.1730
			LO-LC	p=0.8643
	Nitrogen	P=0.00522*	Wild-LC	p=0.0051*
		$F_{2,7}$=12.21	Wild-LO	p=0.0326*
			LO-LC	p=0.3934
X. atropurpureus[O]	Carbon	p=0.263		
		$F_{1,5}$=1.587		
	Nitrogen	p=0.141		
		$F_{1,5}$=3.054		
P. chirus [O]	Carbon	p=7.06e-05 ***		
		$F_{1,5}$=144.2		
	Nitrogen	p=0.00285 **		
		$F_{1,5}$=29.58		
A. purpurescens [C]	Carbon	p=0.787	Wild-LC	p=0.7758
		$F_{2,7}$=0.248	Wild-LO	p=0.9827
			LO-LC	p=0.8826
	Nitrogen	p= 0.00155 **	Wild-LC	p=0.0038*
		$F_{2,7}$=18.73	Wild-LO	p=0.0027*
			LO-LC	p=0.9566
Wild fishes	Carbon	p=0.0258*		
		$F_{3,12}$=4.428		
	Nitrogen	p=0.0355*		
		$F_{3,12}$=3.963		
Lab-Omnivore Diet	Carbon	p=0.666		
		$F_{1,4}$=0.216		
	Nitrogen	p=0.0328*		
		$F_{1,4}$=10.25		
Lab-Carnivore Diet	Carbon	p=0.00319*		
		$F_{3,8}$=11.10		
	Nitrogen	p=0.0526*		
		$F_{3,8}$=3.976		

Supplemental Table S2 Candidate Genes under positive selection in Liver (note there might be more than one GO for each gene)

Uniprot ID	Full Name	Gene Ontology	Omega (dn/ds) Model M0	% coverage
G6PD_TAKRU	Glucose-6-phosphate 1-dehydrogenase	GO:0051156	0.31363	22.26
ACSA_HUMAN	Acetyl-coenzyme A synthetase, cytoplasmic	GO:0019427	0.31965	17.69
FA10A_DANRE	Fatty acid-binding protein 10-A, liver basic	Not found	0.3819	100
G6PT1_HUMAN	Glucose-6-phosphate exchanger SLC37A4	Not found	0.42308	18.18
TIM21_XENLA	Mitochondrial import inner membrane translocase subunit Tim21	GO:0030150	0.66572	48.71
LIPE_HUMAN	Endothelial lipase	GO:0008283	1.00152	34.20

**Supplemental Table S3** Candidate Positively Selected Genes in Pyloric ceca				
Uniprot ID	Full Name	GO	Omega (dn/ds) Model M0	% coverage
TPISB_DANRE	Triosephosphate isomerase B	GO:0006094	0.11128	98.39
RNPL1_MOUSE	Aminopeptidase RNPEPL1	GO:0043171	0.12326	52.64
AQP1_PONAB	Aquaporin-1	GO:0015696	0.38443	76.58
DDHD2_MOUSE	Phospholipase DDHD2	GO:0006888	0.45125	59.94
SER1_DROME	Serine proteases 1/2	GO:0006508	0.48138	89.43
TMC7_CHICK	Transmembrane channel-like protein 7	GO:0006811	0.76999	90.07
CC50B_MOUSE	Cell cycle control protein 50B	GO:0006869	0.82402	94.05
NDUC1_BOVIN	NADH dehydrogenase [ubiquinone] 1 subunit C1, mitochondrial	Not found	0.91442	89.47
PRS27_MOUSE	Serine protease 27	Not found	0.92613	81.1
ELA1_SALSA	Elastase-1	Not found	1.32135	98.73
PA21B_CANLF	Phospholipase A2	GO:0019731	1.33595	86.3
TPA_HUMAN	Tissue-type plasminogen activator	GO:0007596	1.39125	49.47
TBA_XENLA	Tubulin alpha chain	GO:0007017	12.61093	28.51

Supplemental Table S4 Candidate Positively Selected Genes in mid-intestine

Uniprot ID	Full Name	GO	Omega (dn/ds) Model M0	
TPISB_DANRE	Triosephosphate isomerase B	GO:0006094	0.11128	98.39
SDHF2_DANRE	Succinate dehydrogenase assembly factor 2, mitochondrial	GO:0006121	0.11453	100
MA2C1_MOUSE	Alpha-mannosidase 2C1	GO:0006013	0.22929	98.85
ACD11_CHICK	Acyl-CoA dehydrogenase family member 11	Not found	0.28243	80.57
G6PT3_DANRE	Glucose-6-phosphate exchanger SLC37A2	GO:0008643	0.29134	100
SDHB_DANRE	Succinate dehydrogenase [ubiquinone] iron-sulfur subunit, mitochondrial	GO:0009060	0.31831	86.79
PLA2R_MOUSE	Secretory phospholipase A2 receptor	GO:0001816	1.26054	7.13
TRY1_SALSA	Trypsin-1	GO:0007586	1.34063	47.93
NDUA3_MOUSE	NADH dehydrogenase [ubiquinone] 1 alpha subcomplex subunit 3	Not found	1.43125	95.24
TPA_HUMAN	Tissue-type plasminogen activator	GO:0007596	5.42397	47.15

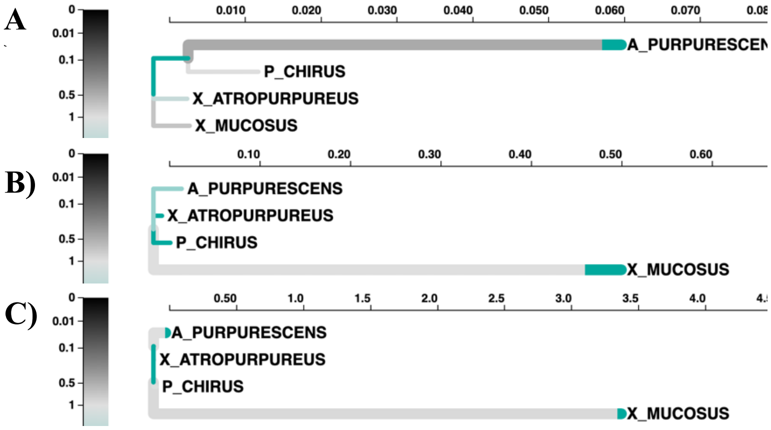

Supplemental Figure S14: An adaptive branch-site random effects likelihood (aBSREL) test for episodic diversification phylogenetic tree constructed for various genes in the pyloric ceca from four prickleback fish species: a) serine protease 27 (PR27), b) tubulin alpha chain (TBA), c) elastase (ELA1). ω is the ratio of nonsynonymous to synonymous substitutions. The color gradient represents the magnitude of the corresponding ω. Branches thicker than the other branches have a $p < 0.05$ (corrected for multiple comparisons) to reject the null hypothesis of all ω on that branch (neutral or negative selection only). A thick branch is considered to have experienced diversifying positive selection.

Supplemental Table S5 Standard length and body mass of wild fishes and fishes fed a carnivore or omnivore diet at the end of the feeding trial

Diet		*X. mucosus*[H]	*X. atropurpureus*[O]	*P. chirus*[O]	*A. purpurescens*[C]
Wild fish	SL (mm)	163.92±32.71<br>n=12	131.72±16.65<br>n=11	100.69±27.68<br>n=16	108.64±17.85<br>n=11
	BM (g)	20.43±10.33<br>n=12	9.05±3.95<br>n=11	5.16±4.42<br>n=16	9.61±5.04<br>n=11
Fish fed Omnivore diet	SL after	159.00±25.17<br>n=8	-	-	99.5±8.78<br>n=6
	BM before	15.59±7.19<br>n=8	-	-	5.99±2.22<br>n=6
	BM after	16.65±8.46<br>n=8	-	-	7.04±2.05<br>n=6
Fish fed Carnivore diet	SL after	156.80±20.91<br>n=5	131.25±15.19<br>n=12	97±9.23<br>n=10	97.29±11.67<br>n=7
	BM before	13.14±5.06<br>n=5	7.54±2.85<br>n=12	3.18±0.72<br>n=10	5.15±2.14<br>n=7
	BM after	15.39±6.13<br>n=5	8.37±3.07<br>n=12	3.89±1.04<br>n=10	6.36±2.84<br>n=7

Wild fishes are separate from fishes fed either an omnivore or carnivore diet in the lab.
BM stands for Body Mass and SL stands for Standard Length

Supplemental Table S6. Growth rate across a four-week feeding trial, and routine metabolic rate of stichaeid fishes fed different diets in the laboratory.

Fish Species	Metabolic Rate (mg O_2 min^{-1} g^{-1})			Growth Rate (% weight gain)		
	Omnivore	Carnivore		Omnivore	Carnivore	
X. mucosus[H]	0.0508 ± 0.0163	0.0369 ± 0.00617	*t*=0.741 *P*=0.478	5.40 ± 5.16 %	16.81 ± 2.57 %	*t*=4.552 *P*=0.001
X. atropurpureus[O]	--	--		--	11.45 ± 2.57 %	
P. chirus[O]	--	--		--	21.68 ± 8.37 %	
A. purpurescens[C]	0.0340 ± 0.00371	0.0413 ± 0.00672	*t*=0.936 *P*=0.373	20.52 ± 12.85 %	22.65 ± 12.02 %	*t*=0.309 *P*=0.763

Note: df=11 for growth comparisons. df=9 for metabolic rate comparisons. Values are mean ± standard error.

Supplemental Results: Relative Gene Expression

We used RNA-seq data of the liver, pyloric ceca and mid-intestine to observe the suites of genes that changed with different diets and how species respond to dietary variation. Note that we are only reporting on pathways relevant to digestion and metabolism of specific nutrient classes (Fig. 4, Table 4). If a cluster is not mentioned, yet depicted in the heatmap, then the genes within that cluster were not directly relevant to digestion and nutrient metabolism.

Liver

There were 11 DEGs when comparing wild fish (WF) and laboratory carnivore diet fish (LC) of *X. atropurpureus* [O], out of which 36% were annotated (Supplemental Figure S14). Cluster 1 (elevated in wild fish) contained genes important for glucose and fatty acid metabolism (Supplemental Table S7). These proteins are important for energy storage, insulin signaling pathway and glucagon signaling pathway. Cluster 2 (elevated in LC fish) contained one unannotated gene.

P. chirus [O] stands out with 302 DEGs when comparing liver gene expression among WF and LC *P. chirus* [O], out of which 14% of genes were annotated (Supplemental Figure S15). Cluster 1 (elevated in wild fish) contains genes involved in cholesterol metabolism (Supplemental Table S4). Cluster 2 (elevated in LC fish) contains genes for lipid metabolism, fatty acid synthesis, and bile acid biosynthesis.

There are 19 DEGs when comparing WF, laboratory omnivore diet fish (LO) and LC *A. purpurescens* [C], out of which 32% of genes were annotated (Supplemental Figure S16). Cluster 1 (elevated in wild fish) consists of genes for cholesterol homeostasis and genes that play a role in controlling the metabolism of fatty acids, specifically glycerophospholipid metabolism and glycerolipid metabolism (Supplemental Table S4).

Pyloric ceca

There were 1226 DEGs when comparing WF to LC *X. atropurpureus* [O], out of which 68.1% were annotated (Supplemental Figure S17). Wild *X. atropurpureus* upregulated genes in Cluster 1 (elevated in wild fish), that were involved in digestive processes for chitin degradation, glycolysis, glycogen catabolic process, glycosaminoglycan biosynthesis, bile acid metabolism, proteolysis, carbohydrate metabolic process, collagen metabolic/catabolic process, pentose phosphate pathway, lipid metabolism, and glutamate biosynthetic process (Supplemental Table

S8). LC *X. atropurpureus* ^{O} upregulated genes in Cluster 2 (elevated in LC fish), that were involved in lipid metabolism.

Like in the liver, *P. chirus* ^{O} showed differing DEGs in comparison to the other species, with only 19 DEGs in the pyloric ceca among WF and LC *P. chirus* ^{O}; 94.7% of the genes were annotated (Supplemental Figure S18). Cluster 1 (elevated in wild fish), featured genes involved in carboxypeptidase activity, proteolysis, and carbohydrate binding (Supplemental Table S8). There were no Cluster 2 (elevated in LC fish) genes in *P. chirus* ^{O} pyloric ceca.

There were 259 DEGs when comparing WF, LC, and LO *A. purpurescens* ^{C}, out of which 62.5% were annotated (Supplemental Figure S19). Cluster 1 (elevated in wild fish) contained genes involved in endopeptidase/trypsin activity and insulin receptor signaling pathway (Supplemental Table S8). Cluster 2 (wild-omnivore genes) contained genes involved in carbohydrate metabolism, bile acid metabolism, cholesterol catabolism, and fatty acid synthesis. Cluster 3 (elevated in the lab genes) contains a large amount of genes, although not directly involved in digestion or metabolism.

Mid-intestine

There were 343 DEGs for W and LC *X. atropurpureus* ^{O}, out of which 83.96% were annotated (Supplemental Figure S20). Cluster 1 (elevated in wild fish) contained genes are involved in glycolysis, cholesterol biosynthesis, lipid metabolism, carbohydrate metabolism, protein metabolism, fatty acid biosynthesis, and pentose phosphate pathway (Supplemental Table S9). Cluster 2 (elevated in LC fish) genes are involved in cholesterol metabolism.

There were 298 DEGs for WF and LC *P. chirus* ^{O}, out of which 90.6% were annotated (Supplemental Figure S21). Cluster 1 (elevated in wild fish) contained 293 of the genes, including those involved in gluconeogenesis, protein deubiquination, creatine metabolic process, and calcium ion transport (Supplemental Table S9). Five genes not directly involved in digestion or nutrient metabolism composed Cluster 2 (elevated in LC fish).

There were 872 DEGs for WF, LC, and LO *A. purpurescens* ^{C}, out of which 82.2% were annotated (Supplemental Figure S22). Cluster 1 (elevated in wild fish) contained genes involved in mannose metabolism, glycogen catabolism, insulin signaling, gluconeogenesis, glucose homeostasis and lipid catabolism (Supplemental Table S9). Cluster 2 (wild-omnivore genes) contained genes involved in bile acid metabolism. Cluster 4 (wild-carnivore genes) contained genes involved in proteolysis. Cluster 5 (carnivore genes) contained genes involved in collagen

catabolism. Cluster 6 (omnivore genes) contained genes involved in cholesterol biosynthesis and glucose metabolism.

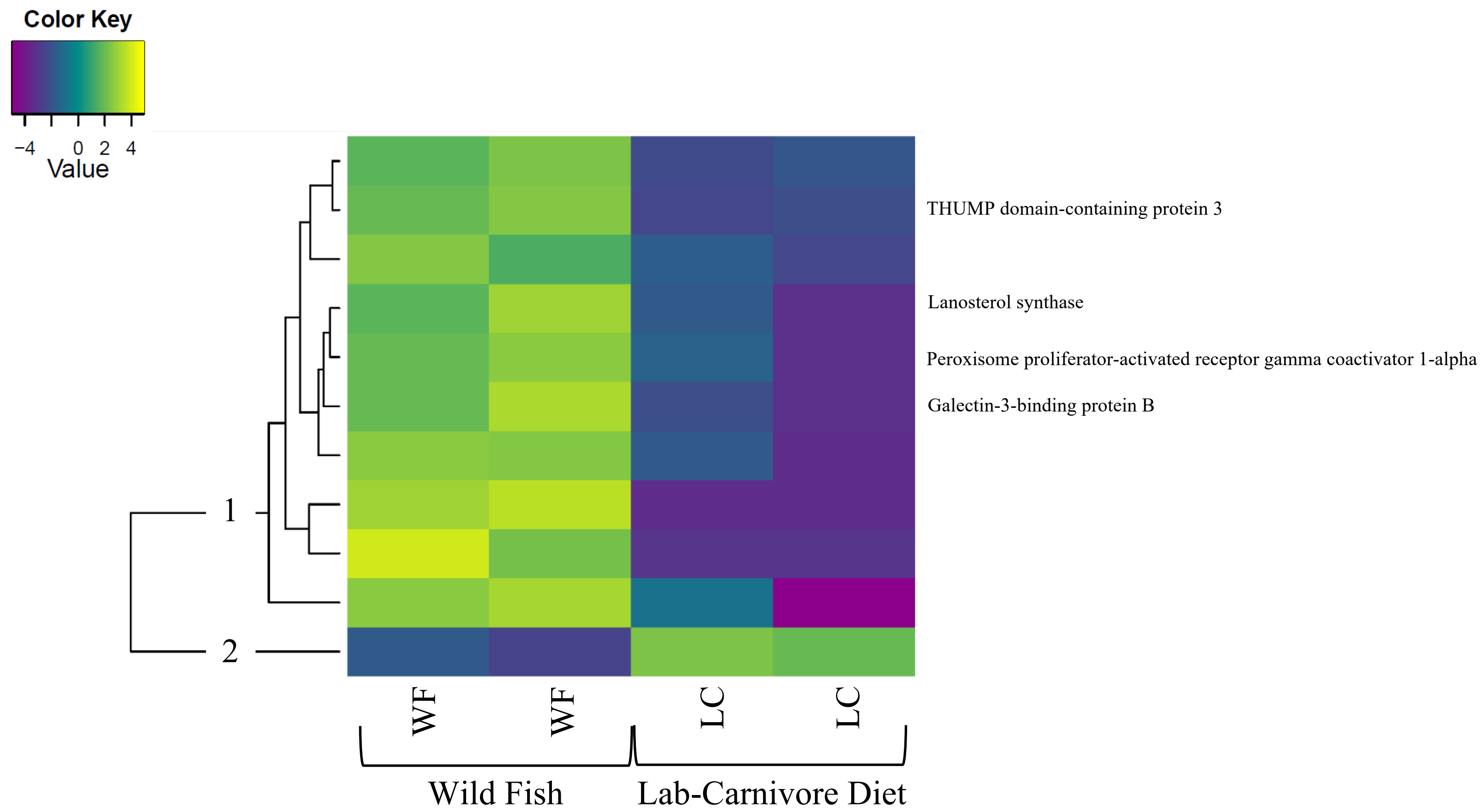

Supplemental Figure S15: Differential gene expression depicted as a heatmap in the liver of *X. atropurpureus* [O]. Yellow indicates elevated relative expression, whereas blue indicates low expression. Each row is a single gene, and genes are clustered in a dendrogram (on left of each heatmap) by similarity of expression patterns. The various clusters of genes are described in Table 4. Each column represents the gene expression in a single tissue from an individual fish, with WF = wild-caught fish, LO = fish fed an omnivore diet in the laboratory (in the case of *X. mucosus*[H] and *A. purpurescens* [C]), and LC = fish fed a carnivore diet in the laboratory.

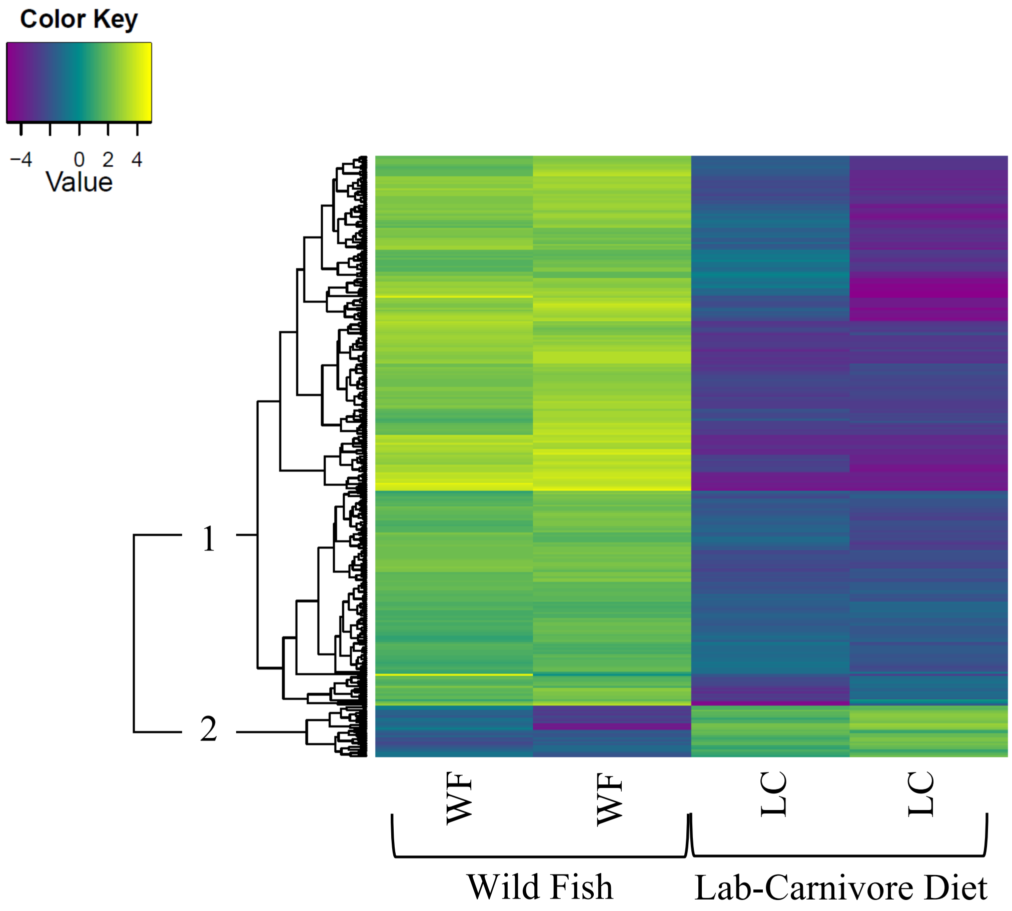

Supplemental Figure S16: As described for Figure S14, but depicting the liver in *P. chirus* [O].

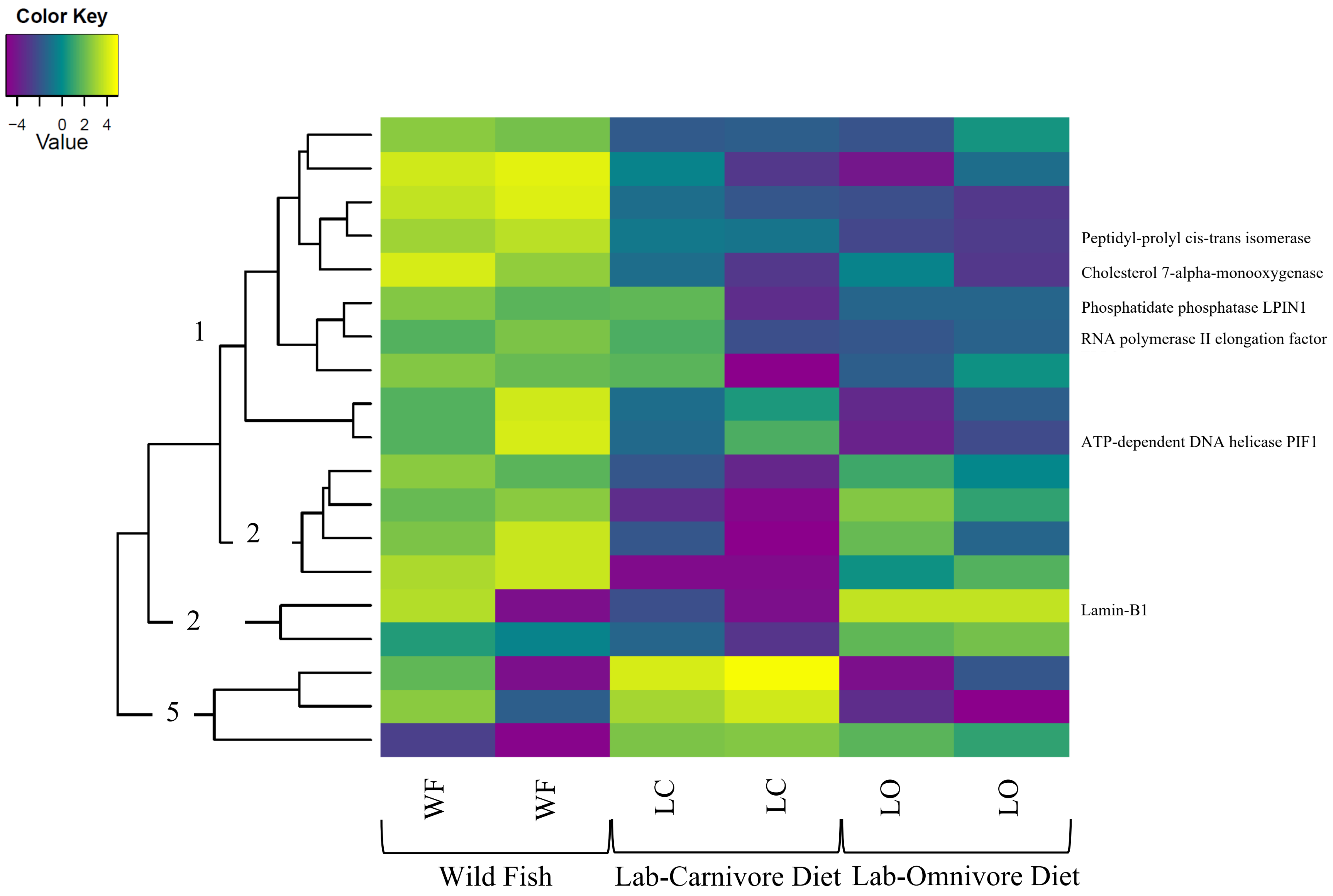

Supplemental Figure S17: As described for Figure S14, but depicting the liver in *A. purpurescens* [C].

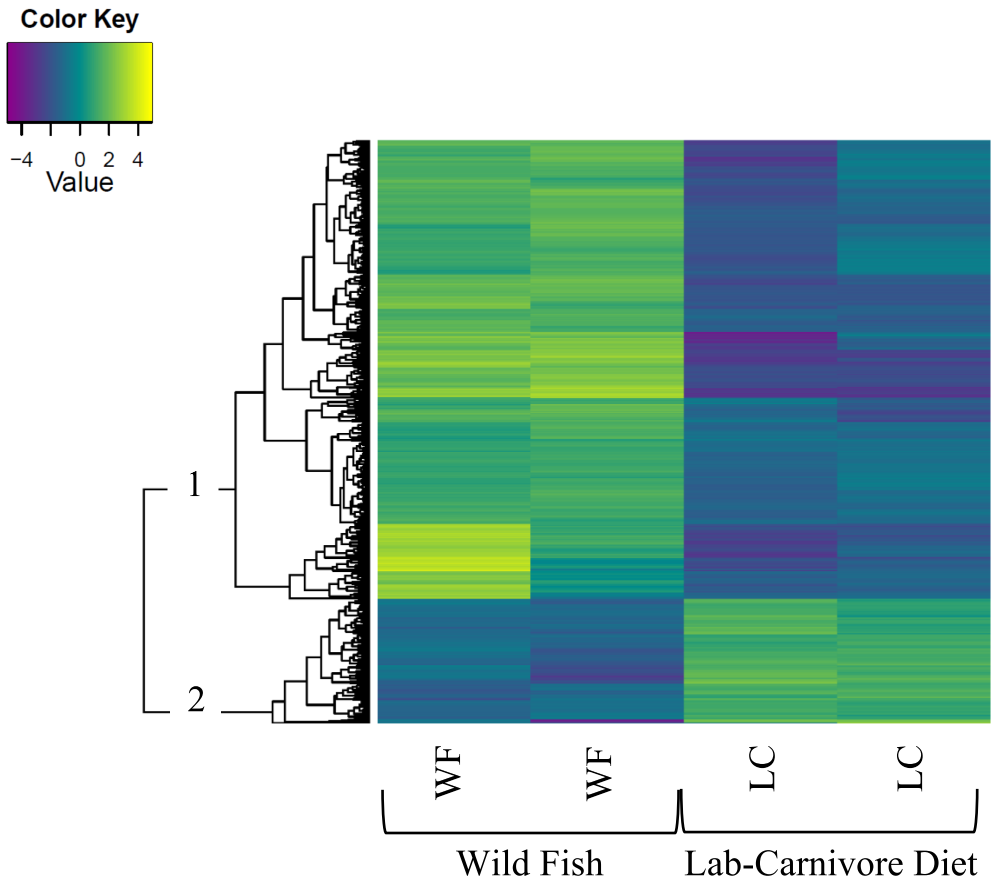

Supplemental Figure S18: As described for Figure S14, but depicting the pyloric ceca in *X. atropurpureus* [O].

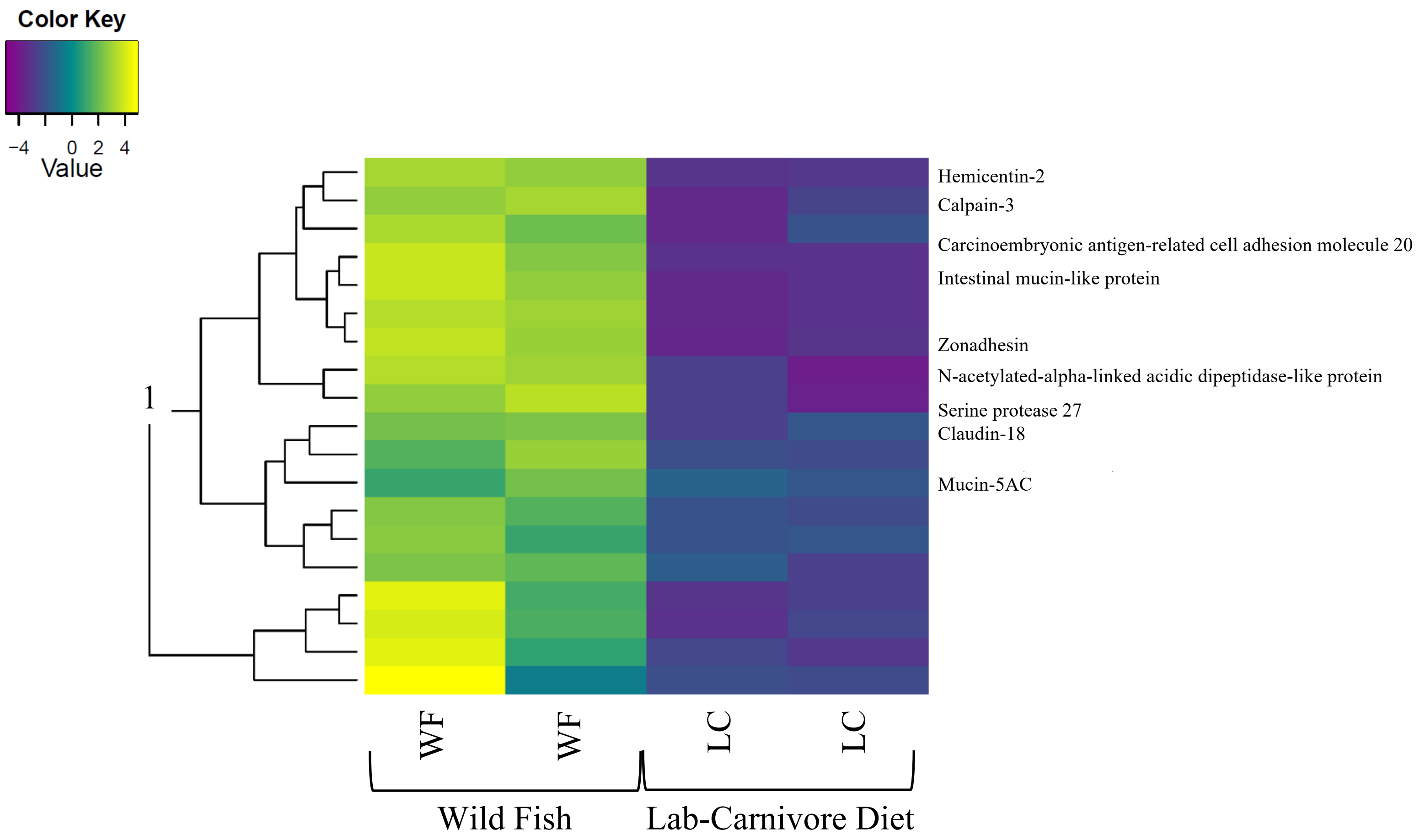

Supplemental Figure S19: As described for Figure S14, but depicting the pyloric ceca in *P. chirus* [O].

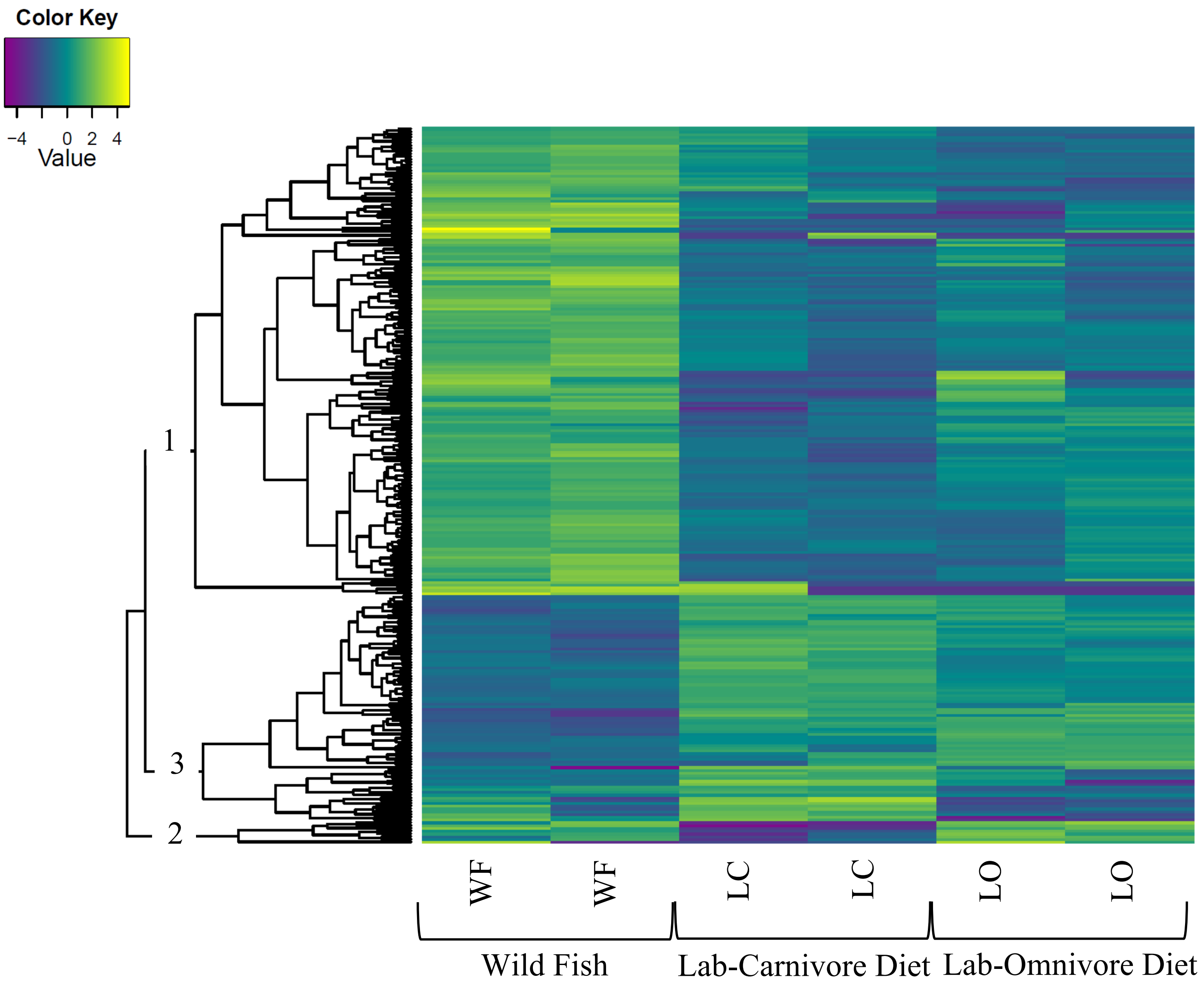

Supplemental Figure S20: As described for Figure S14, but depicting the pyloric ceca in *A. purpurescens* [C].

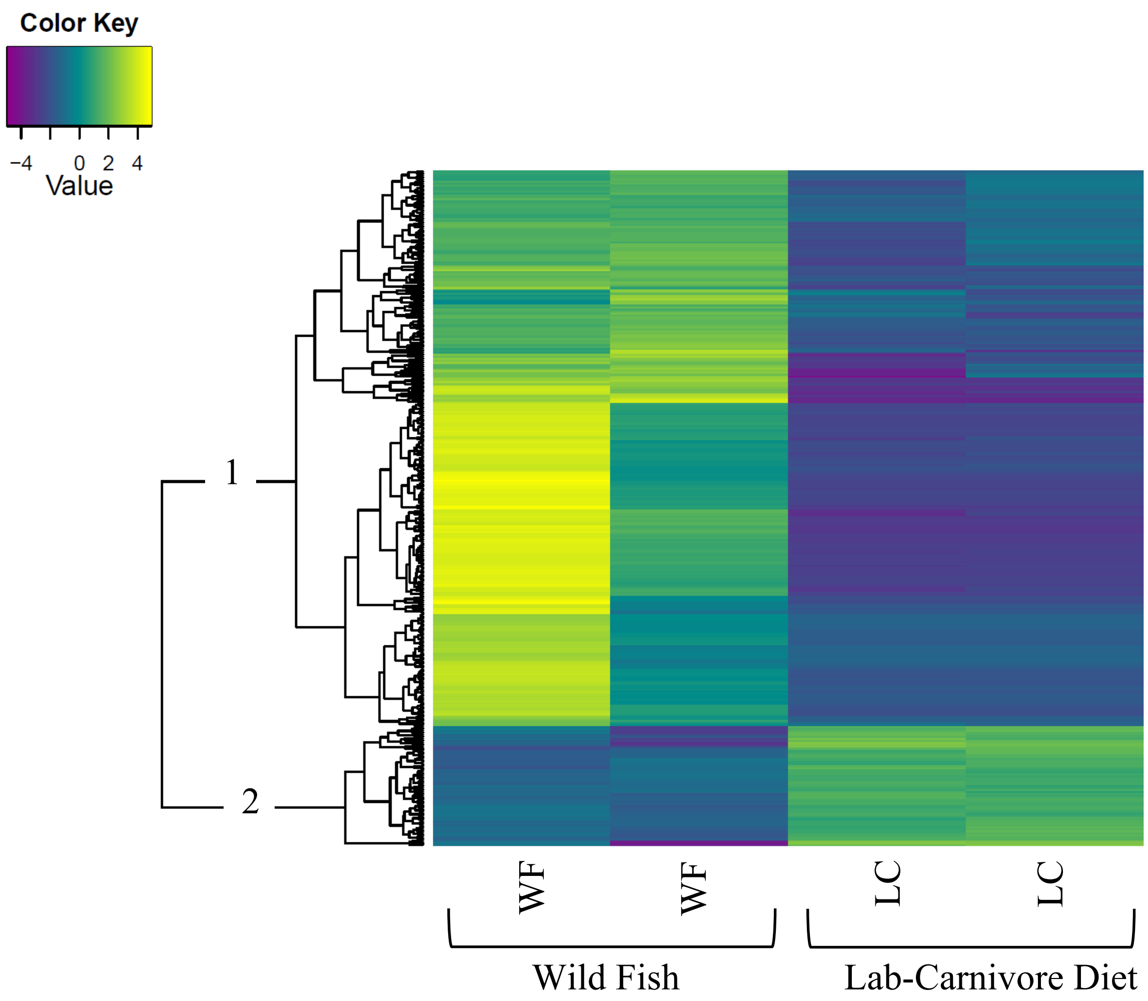

Supplemental Figure S21: As described for Figure S14, but depicting the mid-intestine in *X. atropurpureus* ^{O}.

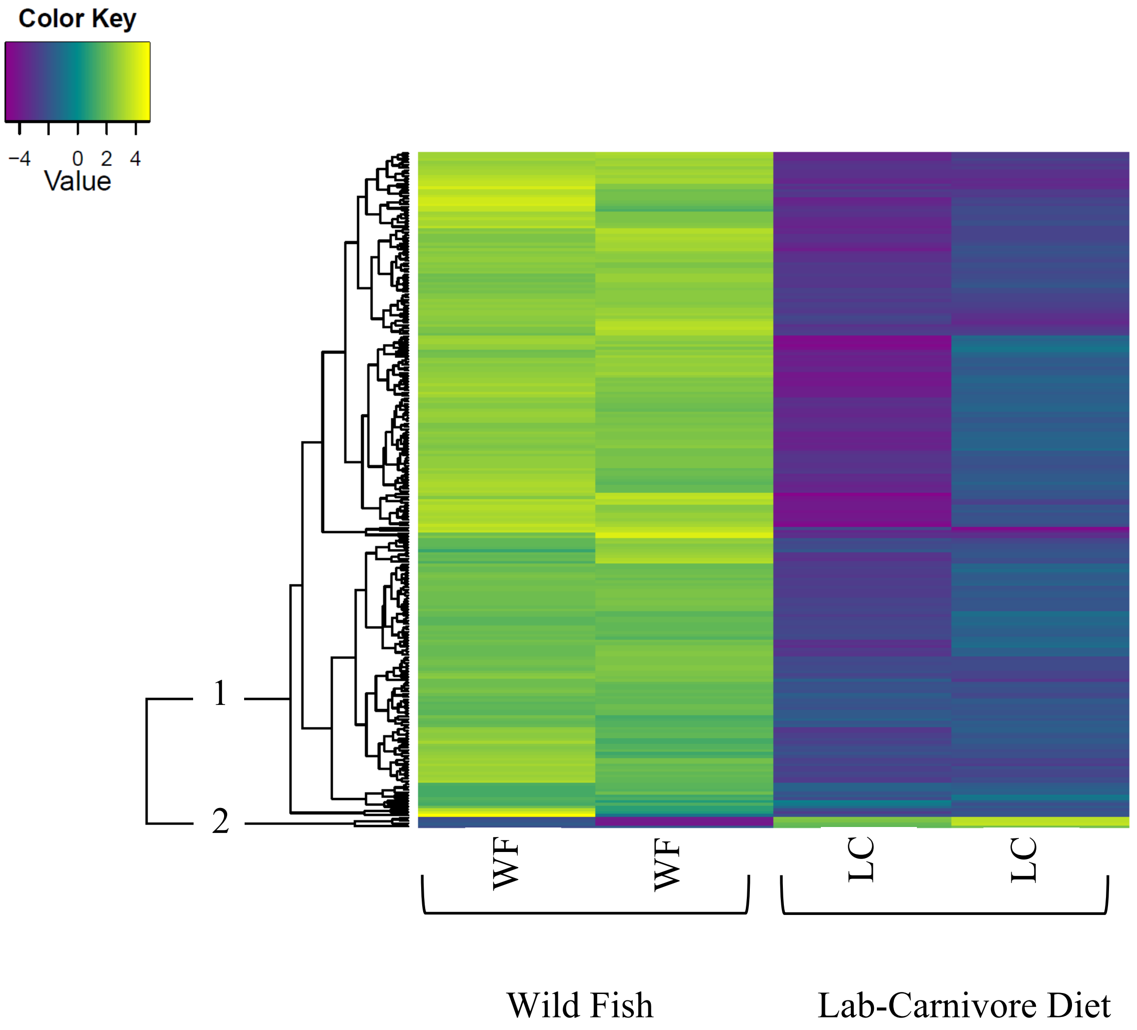

Supplemental Figure S22: As described for Figure S14, but depicting the mid-intestine in *P. chirus* [O].

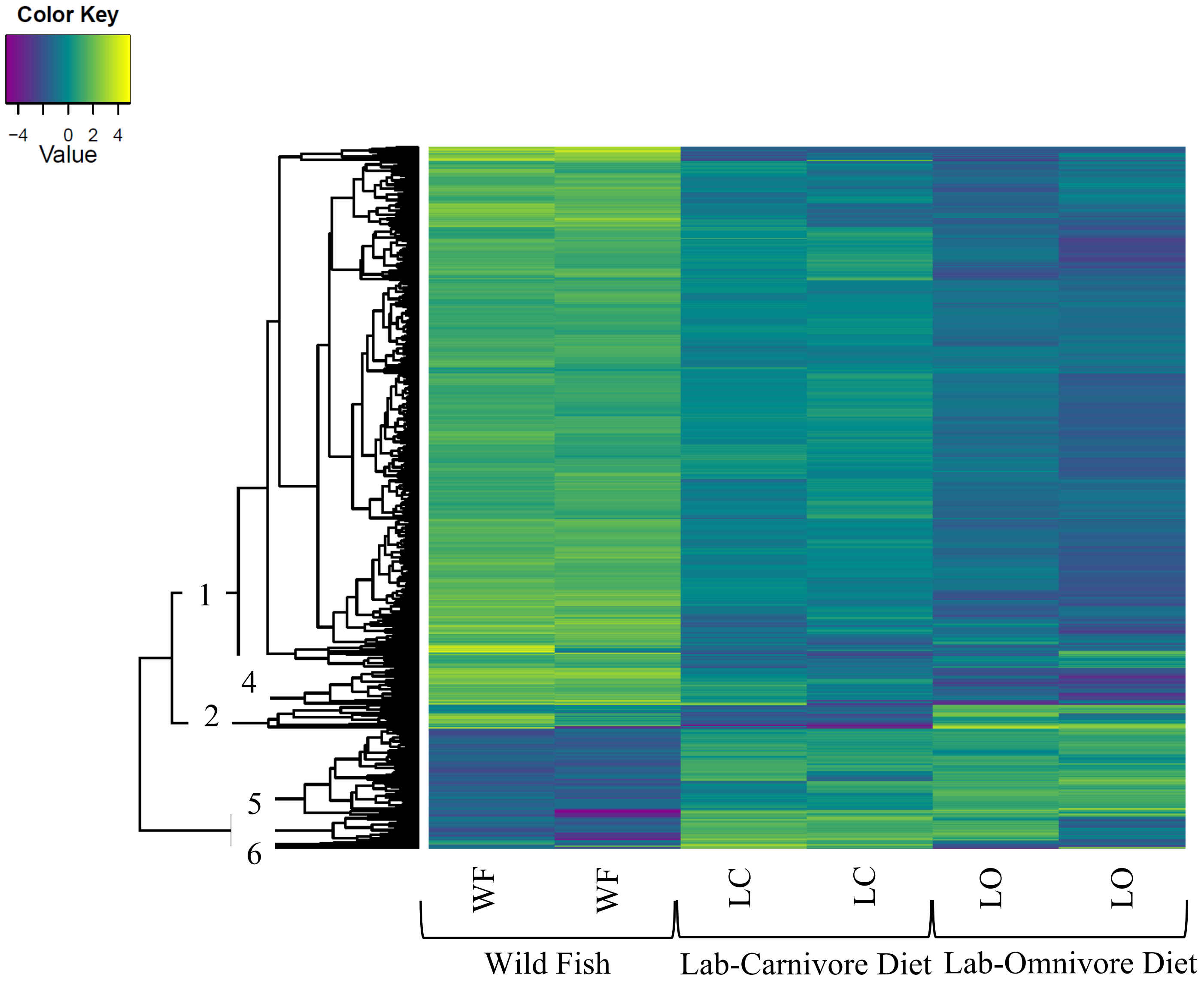

Supplemental Figure S23: As described for Figure S14, but depicting the mid-intestine in *A. purpurescens* [C].

**Supplemental Table S7** Differentially Expressed Genes relevant to metabolism in Liver							
Species	Cluster	Gene	Function	WF fish	LC fish	LO fish	PSG in WF fish
Xiphister atropurpureus [O]	1	Lanosterol synthase	cholesterol biosynthesis	High (++++)	Low (+)	N/A	
	1	peroxisome proliferator-activated receptor gamma coactivator 1-alpha	Coordinates genes involved in glucose and fatty acid metabolism	High (++++)	Low (+)	N/A	
Phytichthys chirus [O]	1	Cholesterol side-chain cleavage enzyme	cholesterol metabolism	High (++++)	Low (+)	N/A	
	2	Apolipoprotein	lipid metabolism	Low (+)	High (+++++)	N/A	
	2	Glucose-6-phosphate 1-dehydrogenase	Pentose phosphate pathway: produces NADPH for fatty acid and nucleic acid synthesis	Low (+)	High (+++++)	N/A	*
	2	Sterol 26-hydroxylase	bile acid biosynthesis	Low (+)	High (+++++)	N/A	
Anoplarchus purpurescens [C]	1	Cholesterol 7-alpha monooxygenase	cholesterol homeostasis	High (+++++)	Low (+)	Low (+)	
	1	phosphatidate phosphatase	controls the metabolism of fatty acids	High (+++++)	Low (++)	Low (++)	

PSG: Positively Selected Gene when comparing sequences (PAML and Datamonkey) among wild-caught fishes of the four prickleback species. Gradient of expression is depicted by plus signs, in that one (+) is low expression to a roughly 5 fold increase (+++++).

**Supplemental Table S8** Differentially Expressed Genes relevant to digestion in Pyloric ceca							
Species	Cluster	Gene	Function	WF fish	LC fish	LO fish	PSG in WF fish
X. atropurpureus O	1	acidic endochitinase SP2	chitin degradation	High (++++)	Low (+)	N/A	
	1	chitinase A	chitin degradation	High (++++)	Low (+)	N/A	
	1	phosphoglycerate kinase	Glycolysis	High (++++)	Low (+)	N/A	
	1	ADP-dependent glucokinase	Glycolysis	High (++++)	Low (+)	N/A	
	1	glucose-6-phosphate isomerase	Glycolysis	High (++++)	Low (+)	N/A	
	1	fructose-bisphosphate aldolase 2	Glycolysis	High (++++)	Low (+)	N/A	
	1	triosephosphate isomerase	Glycolysis	High (++++)	Low (+)	N/A	
	1	glycogen phosphorylase 1	glycogen catabolic process	High (++++)	Low (+)	N/A	
	1	2-phosphoxylose phosphatase	glycosaminoglycan biosynthesis	High (++++)	Low (+)	N/A	
	1	glypican-5	glycosaminoglycan biosynthesis	High (++++)	Low (+)	N/A	
	1	glycerol-3-phosphate dehydrogenase	lipid metabolism	High (++++)	Low (+)	N/A	
	1	gastrotropin	bile acid metabolism	High (++++)	Low (+)	N/A	
	1	Transmembrane protease serine 7	proteolysis	High (++++)	Low (+)	N/A	
	1	Carboxypeptidase M	Proteolysis	High (++++)	Low (+)	N/A	
	1	Puromycin-sensitive aminopeptidase	Proteolysis	High (++++)	Low (+)	N/A	
	1	Pepsin A	Proteolysis	High (++++)	Low (+)	N/A	
	1	L-lactate dehydrogenase	carbohydrate metabolic process	High (++++)	Low (+)	N/A	
	1	Lactoylglutathione lyase	carbohydrate metabolic process	High (++++)	Low (+)	N/A	
	1	Beta-1,4 N-acetylgalactosaminyltransferase 1	carbohydrate metabolic process	High (++++)	Low (+)	N/A	
	1	72 kDa type IV collagenase	collagen catabolic process	High (++++)	Low (+)	N/A	
	1	Prolyl 3-hydroxylase 3	carbohydrate metabolic process	High (++++)	Low (+)	N/A	

	1	Malate dehydrogenase	carbohydrate metabolic process	High (++++)	Low (+)	N/A	
	1	Transketolase	pentose phosphate pathway	High (++++)	Low (+)	N/A	
	1	Collagenase 3	carbohydrate metabolic process	High (++++)	Low (+)	N/A	
	1	Malonyl-CoA-acyl carrier protein transacylas	lipid metabolism	High (++++)	Low (+)	N/A	
	1	Glutamate dehydrogenase 1	glutamate biosynthetic process	High (++++)	Low (+)	N/A	
	2	glycerol-3-phosphate dehydrogenase	cellular lipid metabolic process	Low (++)	High (++++)	N/A	
P. chirus O	1	N-acetylated-alpha-linked acidic dipeptidase-like protein	carboxypeptidase activity	High (+++++)	Low (+)	N/A	
	1	Calpain 9	Proteolysis	High (+++++)	Low (+)	N/A	
	1	Calpain 3	Proteolysis	High (+++++)	Low (+)	N/A	
	1	Serine protease 27	Proteolysis	High (++++)	Low (+)	N/A	*
	1	Galectin 4	Carbohydrate binding	High (++++)	Low (+)	N/A	
A. purpurescens C	1	Serine protease 27	Endopeptidase/trypsin activity	High (++++)	Low (+)	Low (+)	*
	1	Insulin receptor substrate 2A and 2B	Insulin receptor signaling pathway	High (++++)	Low (+)	Low (+)	
	2	Lactase-phlorizin hydrolase	Carbohydrate metabolism	High (++++)	Low (+)	High (+++++)	
	2	Gastrotropin	Bile acid metabolism	High (++++)	Low (+)	High (+++++)	
	2	Cholesterol 7-alpha-monooxygenase	Cholesterol catabolism	High (++++)	Low (+)	High (+++++)	
	2	Fatty acid synthase	Fatty acid synthesis	High (++++)	Low (+)	High (+++++)	

PSG: As described in Table S4. Gradient of expression is depicted by plus signs, in that one (+) is low expression to a roughly 5 fold increase (+++++).

**Supplemental Table S9** Differentially Expressed Genes relevant to digestion in Mid-intestine							
Species	Cluster	Gene	Function	WF fish	LC fish	LO fish	PSG in WF fish
X. atropurpureus [O]	1	Fructose-bisphosphate adolase 2	Glycolysis	High (+++++)	Low (+)	N/A	
	1	Glycogen phosphorylase 1	Glycogen catabolic process	High (+++++)	Low (+)	N/A	
	1	Glucose-6-phosphate isomerase	Glycolysis	High (+++++)	Low (+)	N/A	
	1	Glyceraldehyde-3-phosphate dehydrogenase	Glycolysis	High (+++++)	Low (+)	N/A	
	1	Phosphoglycerate kinase	Glycolysis	High (+++++)	Low (+)	N/A	
	1	Pyruvate kinase	Glycolysis	High (+++++)	Low (+)	N/A	
	1	Lanosterol synthase	Cholesterol biosynthesis	High (+++++)	Low (+)	N/A	
	1	Endothelial lipase	Lipid metabolism	High (+++++)	Low (+)	N/A	
	1	Malate dehydrogenase	Carbohydrate metabolism	High (+++++)	Low (+)	N/A	
	1	L-lactase dehydrogenase	Carbohydrate metabolism	High (+++++)	Low (+)	N/A	
	1	Aminopeptidase	Fatty acid biosynthesis	High (+++++)	Low (+)	N/A	
	1	Prolyl endopeptidase	Fatty acid biosynthesis	High (+++++)	Low (+)	N/A	
	1	Fatty acid synthase	Fatty acid biosynthesis	High (+++++)	Low (+)	N/A	
	1	Transketolase	Pentose phosphate pathway	High (+++++)	Low (+)	N/A	
	2	Apolipoprotein B-100	Cholesterol metabolism	Low (+)	High (++++)	N/A	
Phytichthys chirus [O]	1	Pyruvate carboxylase	Gluconeogenesis	High (+++++)	Low (+)	N/A	
	1	Ubiquitin carboxyl-terminal hydrolase 24	Protein deubiquination	High (+++++)	Low (+)	N/A	
	1	Ubiquitin carboxyl-terminal hydrolase 34	Protein deubiquination	High (+++++)	Low (+)	N/A	
	1	Sodium- and chloride-dependent creatine transporter 1	Ion transport	High (+++++)	Low (+)	N/A	
	1	Sarcoplasmic/endoplasmic reticulum calcium atpase 1	Calcium ion transport	High (+++++)	Low (+)	N/A	
	1	Sarcoplasmic/endoplasmic reticulum calcium atpase 2	Calcium ion transport	High (+++++)	Low (+)	N/A	
A. purpurescens [C]	1	alpha-mannosidase	mannose metabolism	High (++++)	Low (+++)	Low (+++)	

	1	L-fucose kinase	glycogen catabolism	High (++++)	Low (+++)	Low (+++)	
	1	insulin receptor substrate	insulin signaling	High (++++)	Low (+++)	Low (+++)	
	1	insulin receptor substrate 2-B	insulin signaling	High (++++)	Low (+++)	Low (+++)	
	1	pyruvate carboxykinase	gluconeogenesis	High (++++)	Low (+++)	Low (+++)	
	1	phosphoenolpyruvate carboxykinase	glucose homeostasis	High (++++)	Low (+++)	Low (+++)	
	1	lipase	lipid catabolism	High (++++)	Low (+++)	Low (+++)	
	2	Gastropin	bile acid metabolism	High (++++)	Low (+)	High (++++)	
	4	aminopeptidase	proteolysis	High (+++++)	High (++++)	Low (+)	
	5	Stromelysin-3	collagen catabolism	Low (+)	High (++++)	Low (+)	
	6	diphosphomevalonate decarboxylase	cholesterol biosynthesis	Low (+)	Low (+)	High (+++)	
	6	lanosterol synthase	cholesterol biosynthesis	Low (+)	Low (+)	High (+++)	
	6	GDP-D-glucose phosphorylase 1	glucose metabolism	Low (+)	Low (+)	High (+++)	
PSG: As described in Table S4. Gradient of expression is depicted by plus signs, in that one (+) is low expression to a roughly 5 fold increase (+++++).							

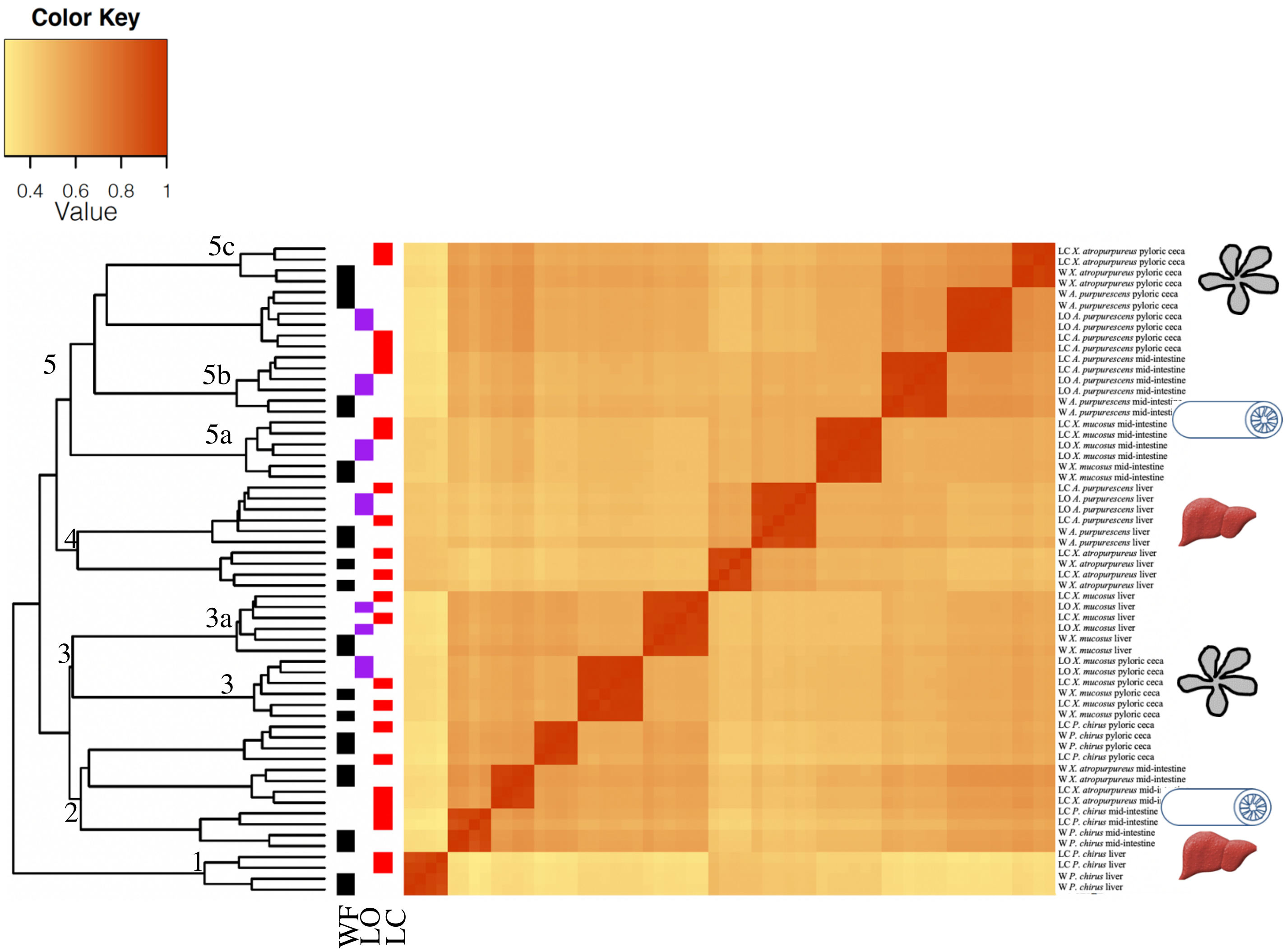

Supplementary Figure S24. Correlation matrix of expressed genes in the liver, pyloric ceca, and mid intestine of four prickleback species caught from the wild (WF), or fed omnivore (LO) or carnivore (LC) diets in the laboratory. Clustering of WF are depicted by black bars in between the dendrogram and the correlation matrix, whereas LO fishes are depicted by purple bars, and LC fishes by red bars. All sample names are depicted on the right side (60 individual tissues in total), with symbols for each tissue type used to emphasize clustering. Correlation matrix created with the Trinity toolkit "PtR" and Pearson correlation as sample distances. We emphasize five distinct clusters: 1: *Phytichthys chirus* ^{O} liver. 2: Mid intestine of *P. chirus* ^{O} and *X. atropurpureus* ^{O}. 3: broken into two clusters, 3a: *X. mucosus*^{H} liver, 3b: *X. mucosus*^{H} pyloric ceca. 4: *Anoplarchus purpurescens* ^{C} and *X. atropurpureus* ^{O} liver. And, 5: broken into three clusters, 5a: *Anoplarchus purpurescens* ^{C} and *X. atropurpureus* ^{O} pyloric ceca, 5b: *A. purpurescens* ^{C} mid intestine, 5c: *X. mucosus*^{H} mid intestine.

Supplemental Discussion

Metabolic Rate

Contrary to our expectation that fishes consuming the carnivore diet in the laboratory would have higher metabolic rates than fishes consuming the omnivore diet, routine metabolic rate did not vary among the species or intra-specifically on the different diets, suggesting that body mass is still one of the main determinants of metabolic rate in fishes, and these fishes are all similar in size in comparison to the range of sizes fishes can attain (Gillooly et al. 2001; Clarke and Johnston 1999; Ikeda 2016). More detailed measures of metabolic rate across longer time scales undoubtedly would show differences in Specific Dynamic Action for fishes consuming the different diets in the laboratory (Secor 2009), but we only measured routine metabolic rate. Given the short period of time over which we measured metabolic rate, our results were possibly influenced by stress and thus, more detailed analyses of metabolic rate in prickleback fishes are needed (Killen et al. 2021).

Clarke A, Johnston NM (1999) Scaling of metabolic rate with body mass and temperature in teleost fish. J Animal Ecol 68 (5):893-905. doi:10.1046/j.1365-2656.1999.00337.

Ikeda T (2016) Routine metabolic rates of pelagic marine fishes and cephalopods as a function of body mass, habitat temperature and habitat depth. J Exp Mar Biol Ecol 480:74-86. doi:https://doi.org/10.1016/j.jembe.2016.03.012

Killen SS, Christensen EAF, Cortese D, Závorka L, Norin T, Cotgrove L, Crespel A, Munson A, Nati JJH, Papatheodoulou M, McKenzie DJ (2021) Guidelines for reporting methods to estimate metabolic rates by aquatic intermittent-flow respirometry. J Exp Biol 224 (18):jeb242522. doi:10.1242/jeb.242522

Gillooly JF, Brown JH, West GB, Savage VM, Charnov EL (2001) Effects of Size and Temperature on Metabolic Rate. Science 293 (5538):2248. doi:10.1126/science.1061967

Secor SM (2009) Specific dynamic action: a review of the postprandial metabolic response. J of Comp Physiol B 179 (1):1-56. doi:10.1007/s00360-008-0283-7

**Supplementary Table S10** Annotated Gene IDs of vectors in PCA plot (Figure 6)	
**Liver**	
Genes associated with *X. mucosus*[H] (towards the left of the PCA plot)	Apolipoprotein B-100
	Probable bifunctional E2/E3 enzyme R795
	CASP8 and FADD-like apoptosis regulator
	Complement factor I
	3-hydroxyanthranilate 3,4-dioxygenase
Genes associated with *X. atropurpureus* [O] (upwards of the PCA plot)	Apolipoprotein B-100
	Very long-chain acyl-CoA synthetase
	Complement factor H-related protein 2
	RNA 3'-terminal phosphate cyclase
	Pyrethroid hydrolase Ces2e
**Pyloric Ceca**	
Genes associated with *X. mucosus*[H] (bottom left of the PCA plot)	Plectin
	Uncharacterized protein 075L
	Sodium channel protein type 4 subunit alpha B
Genes associated with *A. purpurescens* [C] (bottom right of the PCA plot)	ATP-citrate synthase
**Mid-intestine**	
Genes associated with *X. mucosus*[H] (bottom left of the PCA plot)	Agmatinase, mitochondrial
	Serine/threonine-protein kinase 16
	Deoxyribonuclease gamma
	Antizyme inhibitor 1
	Serine/threonine-protein phosphatase 2A 56 kDa regulatory subunit delta isoform
Genes associated with *A. purpurescens* [C] (upwards of the PCA plot)	Peptidyl-prolyl cis-trans isomerase FKBP3
